# Next-generation immunotherapeutic approaches for blood cancers: Exploring the efficacy of CAR-T and cancer vaccines

**DOI:** 10.1186/s40164-025-00662-3

**Published:** 2025-05-17

**Authors:** Kiavash Hushmandi, Abbas Ali Imani Fooladi, Russel J. Reiter, Najma Farahani, Liping Liang, Amir Reza Aref, Noushin Nabavi, Mina Alimohammadi, Le Liu, Gautam Sethi

**Affiliations:** 1 Nephrology and Urology Research Center, Clinical Sciences Institute, Baqiyatallah University of Medical Sciences, Tehran, Islamic Republic of Iran; 2 Applied Microbiology Research Center, Biomedicine Technologies Institute, Baqiyatallah University of Medical Sciences, Tehran, Iran; 3 Department of Cell Systems and Anatomy, UT Health San Antonio, San Antonio, TX 78229 USA; 4 Farhikhtegan Medical Convergence Sciences Research Center, Farhikhtegan Hospital Tehran Medical Sciences, Islamic Azad University, Tehran, Iran; 5 Guangzhou Key Laboratory of Digestive Diseases, Department of Gastroenterology and Hepatology, Guangzhou Digestive Disease Center, Guangzhou First People’s Hospital, School of Medicine, South China University of Technology, Guangzhou, 510180 China; 6 Department of Vitro Vision, DeepkinetiX, Inc, Boston, MA USA; 7 Independent Researcher, Victoria, BC V8V 1P7 Canada; 8 Department of Immunology, School of Medicine, Shahid Beheshti University of Medical Sciences, Tehran, Iran; 9 Integrated Clinical Microecology Center, Shenzhen Hospital, Southern Medical University, Shenzhen, 518000 China; 10 Department of Gastroenterology, Zhujiang Hospital, Southern Medical University, Guangzhou, 510280 China; 11 Department of Pharmacology and NUS Centre for Cancer Research (N2CR), Yong Loo Lin School of Medicine, National University of Singapore, Singapore, 117600 Singapore

**Keywords:** Leukemias, Immunotherapy, Cancer vaccines, CAR-T cell therapy, Immune evasion

## Abstract

Recent advancements in immunotherapy, particularly Chimeric antigen receptor (CAR)-T cell therapy and cancer vaccines, have significantly transformed the treatment landscape for leukemia. CAR-T cell therapy, initially promising in hematologic cancers, faces notable obstacles in solid tumors due to the complex and immunosuppressive tumor microenvironment. Challenges include the heterogeneous immune profiles of tumors, variability in antigen expression, difficulties in therapeutic delivery, T cell exhaustion, and reduced cytotoxic activity at the tumor site. Additionally, the physical barriers within tumors and the immunological camouflage used by cancer cells further complicate treatment efficacy. To overcome these hurdles, ongoing research explores the synergistic potential of combining CAR-T cell therapy with cancer vaccines and other therapeutic strategies such as checkpoint inhibitors and cytokine therapy. This review describes the various immunotherapeutic approaches targeting leukemia, emphasizing the roles and interplay of cancer vaccines and CAR-T cell therapy. In addition, by discussing how these therapies individually and collectively contribute to tumor regression, this article aims to highlight innovative treatment paradigms that could enhance clinical outcomes for leukemia patients. This integrative approach promises to pave the way for more effective and durable treatment strategies in the oncology field. These combined immunotherapeutic strategies hold great promise for achieving more complete and lasting remissions in leukemia patients. Future research should prioritize optimizing treatment sequencing, personalizing therapeutic combinations based on individual patient and tumor characteristics, and developing novel strategies to enhance T cell persistence and function within the tumor microenvironment. Ultimately, these efforts will advance the development of more effective and less toxic immunotherapeutic interventions, offering new hope for patients battling this challenging disease.

**Graphical Abstract:**

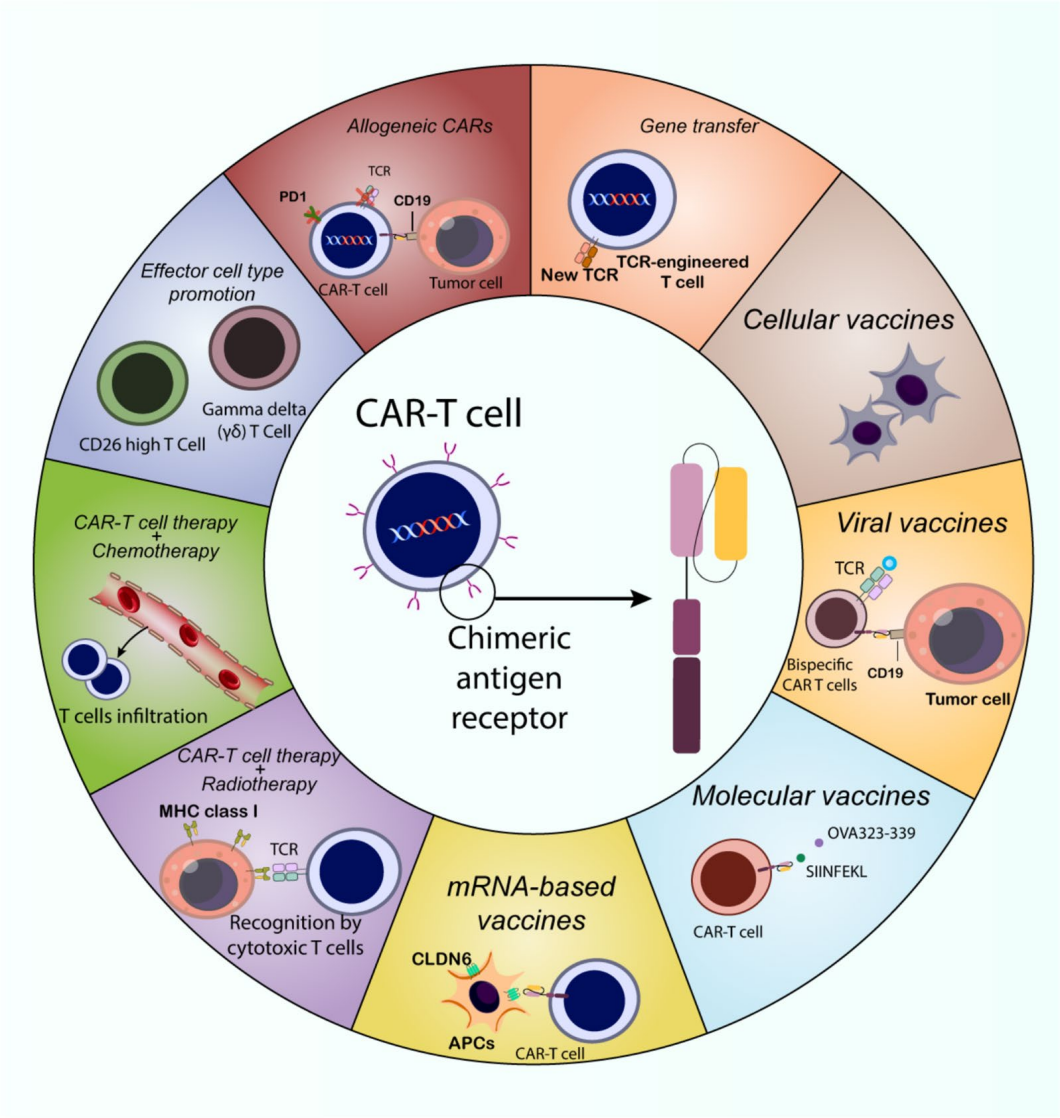

## Introduction

**Fig. 1:**
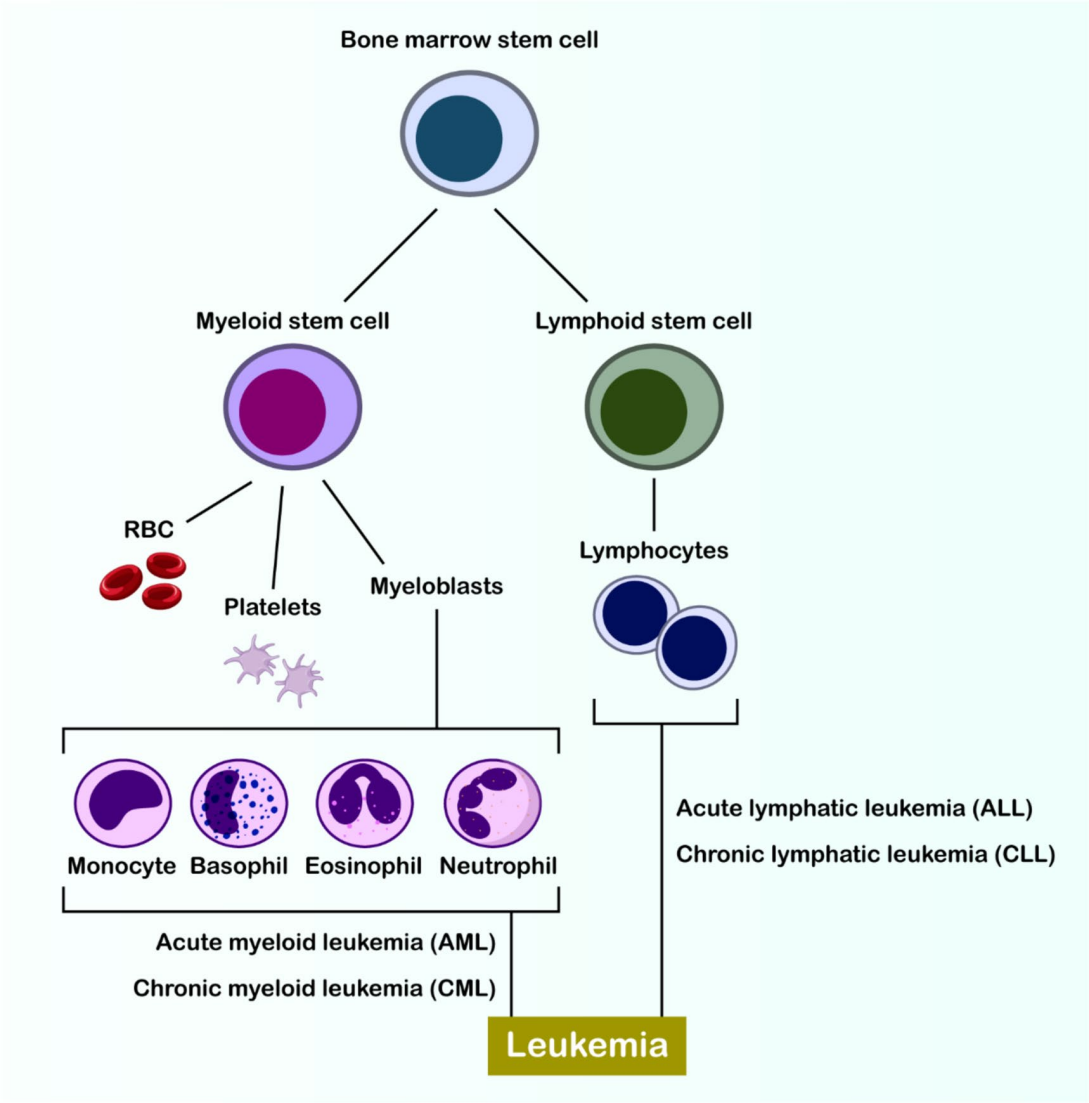
Hematopoiesis and the origins of leukemia. The diagram illustrates the differentiation of a bone marrow stem cell into myeloid and lymphoid lineages, highlighting the mature blood cells derived from each. The figure further depicts the classification of leukemias based on the affected cell lineage and the stage of maturation, categorizing them into acute and chronic myeloid leukemia (AML, CML) arising from myeloid precursors, and acute and chronic lymphocytic leukemia (ALL, CLL) originating from lymphoid precursors. This schematic provides a foundational understanding of the cellular basis of various hematological malignancies collectively termed leukemia

Blood-forming system cancers, known as hematological malignancies, represent a complex array of diseases that affect multiple bodily organs and systems. These disorders encompass several categories including both acute and chronic forms of leukemia (Fig. 1), various lymphomas, multiple myeloma (MM), myelodysplastic syndromes (MDS), and myeloproliferative neoplasms (MPNs) [1]. The significant diversity found in leukemias creates substantial obstacles for immune-based treatments such as CAR-T cell therapy and vaccination approaches. This complexity is evident in how antigens like CD33, CD123, and CLL-1 are expressed differently across AML patient populations and disease subtypes. The effectiveness of CAR-T cell treatments can be compromised when leukemic cells modify or eliminate their target antigens, a process termed antigen escape. Scientists are now investigating CAR-T cells that can target multiple antigens concurrently to enhance immune responses and prevent escape mechanisms. The diverse antigen presentation in AML also presents challenges for vaccine development, as successful immunization requires consistent antigen expression. Potential solutions include developing vaccines targeting multiple antigens or creating patient-specific approaches [2, 3]. In the classification of lymphomas, there are two primary categories: Hodgkin lymphoma (HL) and non-Hodgkin lymphoma (NHL). NHL, which occurs more frequently, develops from lymphocytes at different developmental stages. Various NHL forms, including diffuse large B-cell lymphoma (DLBCL), mantle cell lymphoma, and follicular lymphoma (FL), exhibit characteristics specific to their cell of origin. HL, though less prevalent, stands apart due to its distinctive histological, immunophenotypic, and clinical characteristics, and is subdivided into classical HL and nodular lymphocyte-predominant HL [4, 5]. MM, MDS, and MPN are commonly observed in elderly individuals. Multiple myeloma, a notable hematological malignancy, constitutes 10% of all malignancies and frequently initiates with asymptomatic precursor conditions, such as indeterminate monoclonal gammopathy or smoldering multiple myeloma. MDS is a clonal disorder characterized by ineffective hematopoiesis and has the potential to progress to AML [1]. Despite significant progress in chemotherapy, radiotherapy, and targeted therapies leading to improved overall response rates in cancer patients, recurrence and treatment resistance continue to pose major challenges. Conventional multi-drug chemotherapy is essential for addressing hematologic malignancies; however, molecular heterogeneity necessitates the formulation of innovative treatment strategies owing to the diverse characteristics of these cancers [6].

Cancer immunosurveillance is a complex mechanism in which the immune system utilizes both innate and adaptive cells and molecules to detect and eradicate cancer cells [7]. However, external factors can affect this process, referred to as “extrinsic immune stress.” These factors can either hinder tumor growth by boosting the immune response or promote tumor progression by altering the tumor’s immunogenicity or suppressing anti-tumor immunity [7, 8]. One theory suggests that tumors can evade immune surveillance and remain dormant for extended periods before reemerging, a process known as “immune editing,” which involves phases of equilibrium and senescence [9]. Ultimately, as variants with diminished immunogenicity arise and the host's immune response deteriorates, cancer cells can entirely circumvent immunological surveillance [10]. Cancer cells utilize a variety of strategies to suppress the body's innate defenses throughout all phases of the anti-tumor immune response [11].

The “Cancer-Immunity Cycle,” a complex process that involves the activation of an anti-tumor immune response, can be disrupted in individuals with cancer [12]. Any disruption in these steps leads to the breakdown of the cycle, allowing cancer cells to evade the immune system [13]. Immunotherapy is an innovative strategy that leverages the body's immune system to revive its ability to fight tumors. This groundbreaking treatment approach, developed over several decades, has shown considerable promise in treating cancer patients [14, 15]. Hematological malignancies, in particular, possess distinctive characteristics that make them especially amenable to immunotherapy [16]. In the hematopoietic system, immune cells and cancer cells are in constant interaction, creating an environment that supports immune surveillance. Additionally, the common cellular origin of both malignancies and the immune system can make these cancers immunostimulatory. However, this dynamic can also lead to weakened or inhibited immune responses [1].

Various forms of immunotherapy have demonstrated remarkable success in treating specific leukemia types, particularly acute lymphoblastic leukemia and chronic lymphocytic leukemia. The implementation of CD19-targeted CAR-T cell therapy has marked a breakthrough in managing resistant or recurring ALL cases. Patients with CLL have experienced positive outcomes from specialized CAR-T treatments, including axicabtagene ciloleucel (axi-cel) and lisocabtagene maraleucel (liso-cel), which are specifically designed to target their disease characteristics. Ongoing research in leukemia vaccine development shows potential for addressing immune evasion challenges, especially in CLL cases [17, 18].

The fundamental principle of cancer vaccines involves activating the body's immune defenses to identify and eliminate malignant cells. These immunological interventions can be customized to recognize specific cancer cell markers, enabling precise targeting. Their potential to establish lasting immunity offers a promising strategy for preventing cancer recurrence. For patients who don’t respond to conventional treatments, cancer vaccines can trigger comprehensive and sustained T-cell responses. In contrast, CAR-T cell therapy involves modifying patients’ own T cells to enhance their cancer-fighting capabilities. This innovative approach has proven particularly effective against certain hematological malignancies, functioning as a persistent therapeutic agent that provides ongoing protection against disease recurrence. CAR-T therapy often serves as a crucial alternative when standard treatments fail, particularly in cases of aggressive or treatment-resistant cancers. Both therapeutic strategies harness immune system mechanisms to provide targeted, durable treatment outcomes. The choice between these approaches typically depends on multiple factors, including cancer type, patient status, and the need for alternative solutions when conventional treatments prove ineffective [19, 20].

Cancer immunotherapy is progressing rapidly utilizing different strategies that tap into the natural capabilities of immune system. However, these approaches come with their own set of challenges that need to be overcome. This article seeks to provide readers with up-to-date knowledge on two significant immunotherapeutic options: cancer vaccines and CAR-T cell therapy. We describe how these therapies work, review their clinical results in treating blood cancers, and discuss anticipated advancements that could enhance their efficacy and broaden their use in cancer treatment. This comprehensive description aims to highlight the potential of these therapies in revolutionizing cancer care, while also addressing the hurdles that must be navigated to maximize their therapeutic impact.

## The landscape of leukemia immunotherapy

**Fig. 2:**
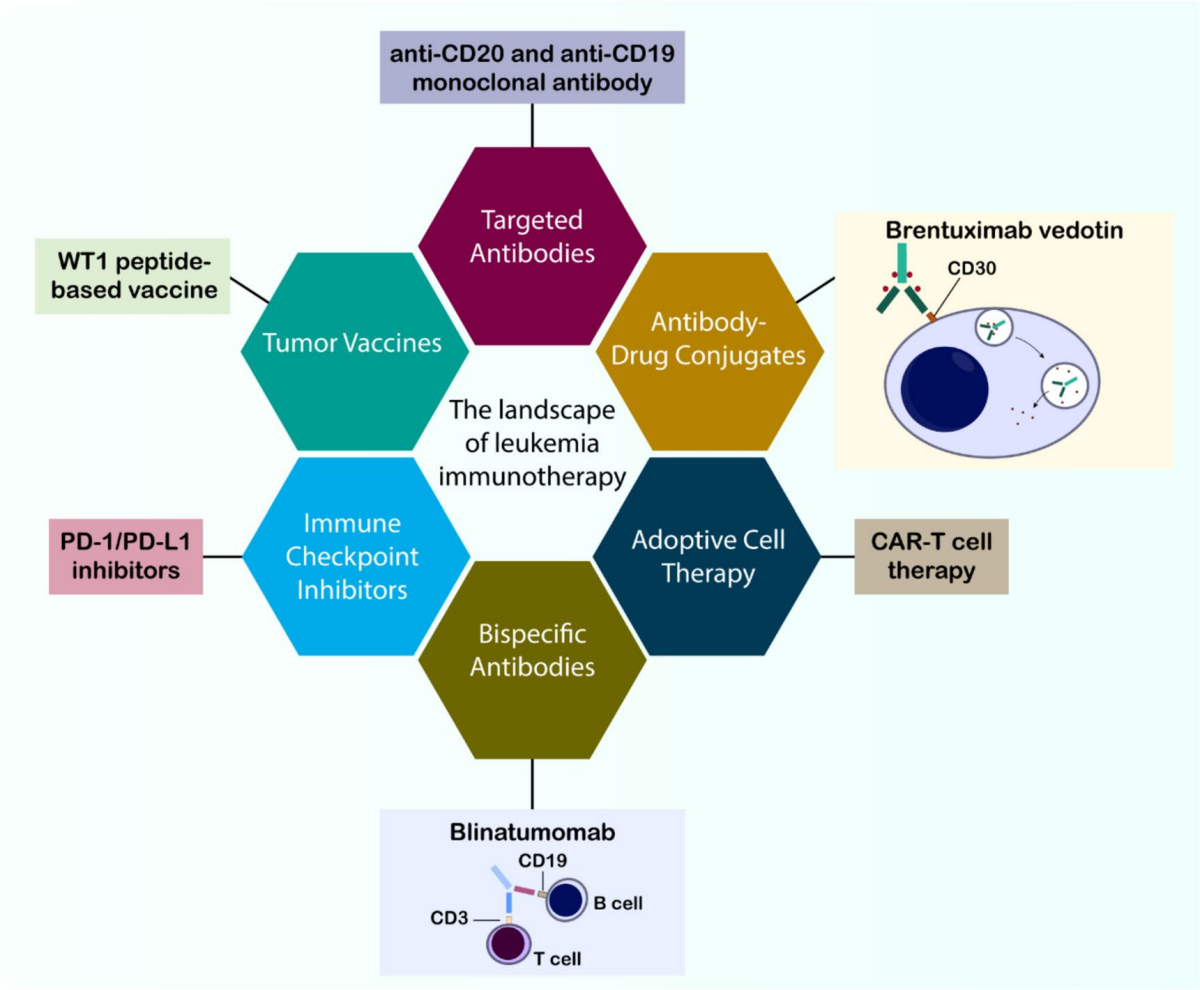
Overview of Immunotherapeutic Strategies in Leukemia. The diverse landscape of current immunotherapeutic approaches for treating leukemia include cancer vaccines (e.g., WT1 peptide-based vaccine), targeted antibodies (e.g., anti-CD20 monoclonal antibody), antibody–drug conjugates (ADCs) exemplified by Brentuximab vedotin targeting CD30, adoptive cell therapy encompassing CAR-T cell therapy, bispecific antibodies such as Blinatumomab bridging CD19 on B cells and CD3 on T cells, and immune checkpoint inhibitors targeting the PD-1/PD-L1 axis

Immunotherapy offers an effective treatment option for hematologic malignancies, particularly leukemias, providing patients with the potential for a cure through various approaches (Fig. 2). These strategies involve a range of tactics, as outlined below.

Antibodies such as Rituximab, a pioneering anti-CD20 monoclonal antibody, directly aim at specific proteins in cancer cells and instigate their elimination [21]. This method has become fundamental in the management of B-cell malignancies, including DLBCL and FL [22]. Recent antibodies, such as tafasitamab and daratumumab, target unique antigens on B-cell malignancies [23, 24].

Bispecific antibodies (BsAbs), often referred to as “bridges,” serve a vital function in cancer treatment by linking T cells to cancer cells, thus facilitating T cell-mediated tumor eradication. This mechanism is essential for the immune response, enabling the immune system to directly identify and eradicate cancer cells. A prime example of this approach is Blinatumomab, a bispecific T-cell engager (BiTE) antibody. BiTE antibodies are a unique class of therapeutic antibodies designed to bind to two different targets simultaneously [25]. Blinatumomab is an antibody engineered to target CD19, a protein present in B cells, including malignant B cells in ALL, and CD3, a protein located in T cells. By connecting these two targets, Blinatumomab brings T cells near cancer cells, thereby facilitating the destruction of the cancer cells by T cells. This bispecific antibody has demonstrated considerable efficacy in treating refractory or relapsed precursor B-cell ALL, a condition marked by the failure of standard therapies or recurrence after initial treatment [26]. The application of blinatumomab in this context has demonstrated encouraging outcomes, providing optimism for patients confronting this difficult condition. This success underscores the potential of bispecific antibodies and similar immunotherapeutic approaches in treating hematologic malignancies, with the possibility of extending their efficacy to other cancer types. Continued research in this domain is anticipated to result in the creation of novel bispecific antibodies aimed at diverse antigens, thus broadening the spectrum of cancers that may benefit from this pioneering therapeutic approach [1, 26].

ADCs represent a significant advancement in cancer therapy. These complex molecules combine the precision of antibodies with the cytotoxic power of drugs, enabling targeted delivery directly to cancer cells [27]. This approach allows for the focused administration of potent drugs to malignant cells, minimizing damage to healthy tissues—a common issue with traditional chemotherapy [28]. Brentuximab vedotin is an FDA-approved ADC used to treat relapsed HL and anaplastic large-cell lymphoma. It is specifically designed to target CD30, a protein often overexpressed on the surface of these cancerous cells [29, 30]. The antibody portion of Brentuximab vedotin binds to CD30, triggering the cancer cell to internalize the entire ADC. Once inside the cell, the cytotoxic drug is released, causing the cancer cell to undergo death [1]. The success of Brentuximab vedotin in treating these lymphoma types marks a significant milestone in the field of ADCs. ADCs have proven effective in delivering cytotoxic agents directly to cancer cells, resulting in enhanced patient outcomes. This progress has paved the way for the development of additional ADCs targeting different antigens and utilizing various cytotoxic drugs. However, challenges such as drug resistance, off-target effects, and the identification of suitable targets remain. Research and clinical trials seek to augment the efficacy and safety of ADCs, broadening their application in diverse cancers and potentially enhancing patient quality of life [31, 32].

CD123-CAR-NK cells have shown significant potential in targeting CD123-positive AML cells while demonstrating a safer profile compared to CAR-T cells. Their "off-the-shelf" nature and reduced risk of graft-versus-host disease make them a compelling option for immunotherapy. Similarly, CD33-CAR-NKT cells have been engineered to target CD33-positive AML cells effectively, with preclinical and early clinical studies highlighting their antitumor activity and improved safety profile [33, 34].

In the field of cancer immunotherapy, therapeutic vaccines have emerged as a promising intervention method. These vaccines introduce specific cancer-related antigens that enable the immune system to detect and combat malignant cells [35, 36]. This process triggers an immunological response, leading to the development of specialized T cells programmed to destroy cells expressing these particular antigens. A notable example in current research is the development of Wilms’ Tumor 1 (WT1) peptide vaccines for treating myelodysplastic syndrome (MDS) patients [37, 38]. Initially identified in Wilms’ tumor, the WT1 gene shows elevated expression in multiple cancer types, including both leukemia and MDS. The vaccine’s mechanism relies on the immune system's ability to recognize the introduced WT1 peptide as foreign, subsequently generating WT1-specific T cells that target cells with high WT1 protein expression. While still under investigation, preliminary findings from WT1 peptide vaccine studies in MDS patients show promising results, potentially offering an alternative treatment option, particularly for individuals unsuitable for conventional therapies. Scientists continue to address various challenges, including optimizing immune response durability, reducing adverse effects, and establishing ideal vaccination protocols. Despite these ongoing challenges, this vaccine development represents significant progress in cancer immunotherapy research, potentially offering new treatment possibilities for MDS and other malignancies [39].

The introduction of immune checkpoint inhibitors (ICIs), particularly those targeting the PD-1/PD-L1 pathway, has transformed cancer immunotherapy strategies. These therapeutic agents enhance the immune system's anti-cancer response by blocking regulatory pathways that typically limit T cell activity [40]. The PD-1/PD-L1 axis serves as a critical regulatory mechanism that helps maintain self-tolerance in normal physiological conditions. Under typical circumstances, PD-1 and PD-L1 interactions prevent autoimmune responses by moderating T-cell reactivity in tissues. Unfortunately, tumor cells frequently hijack this protective mechanism by upregulating PD-L1 expression, which engages with PD-1 receptors on T lymphocytes and inhibits their anti-tumor capabilities. Beyond the established PD-1/PD-L1 checkpoint, researchers are increasingly investigating alternative immune checkpoints such as LAG-3 and TIGIT for treating blood cancers. These additional regulatory molecules contribute to immunosuppression within the tumor microenvironment, and blocking them shows promise for restoring anti-cancer immune responses. Ongoing clinical investigations of therapies targeting these novel checkpoints may expand immunotherapeutic options for hematological malignancies [1].

The mechanism of PD-1/PD-L1 antagonists involves blocking the interaction between these molecules, enabling T cells to effectively recognize and destroy cancer cells, marking a distinct departure from traditional cancer treatments like chemotherapy and radiation. These agents have demonstrated particular success in treating B-cell lymphomas (BCLs), with exceptional outcomes in Hodgkin lymphoma (HL). The effectiveness in HL is attributed to its characteristic Reed-Sternberg cells, which are enlarged cancerous lymphocytes exhibiting elevated PD-L1 expression. While these inhibitors represent a significant therapeutic advancement, challenges such as immune-related side effects and treatment resistance persist [40]. Current scientific efforts concentrate on improving treatment efficacy, reducing adverse effects, and discovering biomarkers that can predict treatment outcomes.

The introduction of PD-1/PD-L1 inhibitors has revolutionized cancer immunotherapy, though ongoing research aims to optimize their clinical application. Scientists are investigating biomarkers like PD-L1 expression levels to better predict patient responses and customize treatment approaches [41].

Recent research has explored combining PD-1/PD-L1 inhibitors with other therapeutic modalities. A 2024 investigation demonstrated promising results when combining PD-1 inhibitors with chemotherapy and targeted treatments, showing enhanced response rates and potential solutions to resistance issues [42].

To summarize, PD-1/PD-L1 inhibitors have profoundly transformed the cancer treatment paradigm, yet ongoing research is focused on overcoming their limitations and improving their clinical application. By identifying predictive biomarkers and investigating combination strategies, researchers aim to enhance the efficacy of these therapies and improve patient outcomes in the ever-evolving field of cancer immunotherapy.

CAR-T cell therapy involves genetic modification of T cells to express chimeric antigen receptors targeting specific cancer antigens [43]. This approach has shown remarkable success in treating various blood cancers, including refractory or relapsed ALL, CLL, NHL, and MM. Following the first successful B-cell lymphoma treatment in 2008, the FDA approved commercial CAR-T products in 2017. Meanwhile, allogeneic hematopoietic stem cell transplantation (allo-HSCT) remains crucial for certain blood cancers [44]. Despite its five-decade history, allo-HSCT continues to play a vital role in blood cancer treatment, offering potential complete cure, though risks like graft-versus-host disease and infection susceptibility remain significant. Modern immunotherapy encompasses various approaches, including immune checkpoint inhibitors, CAR-T cells, cancer vaccines, and bispecific antibodies, each designed to enhance the immune system's cancer-fighting capabilities [45–49]. CAR-T therapy has evolved from initially targeting CD19 in B-cell malignancies to treating various leukemia types. Recent data shows impressive complete remission rates of 70–90% in younger patients with relapsed/refractory B-cell ALL, and 50–80% response rates in large B-cell lymphoma. Novel CAR-T therapies targeting CD7 and CD5 show promise for T-cell leukemias [50, 51]

These advances in immunotherapy, combined with established treatments like allo-HSCT, suggest an encouraging future for blood cancer treatment as research continues to enhance our understanding of immune system-cancer interactions.

## Cancer vaccines: mechanisms and development

The success of vaccines in preventing infectious diseases stands as one of the major medical achievements of the twentieth century. The core principles of vaccination go beyond mere prevention. With a deeper understanding of the immune system, therapeutic vaccines have been developed to treat infections, and several of these vaccines have shown promising results in advanced clinical trials [52]. These advancements facilitate the creation of more efficacious vaccines. The notion of employing vaccines for the treatment of pre-existing cancers has a longstanding history, with William Coley as a trailblazer in the early twentieth century. He was one of the first to use injections of killed bacteria (Streptococcus and Serratia) as a treatment for tumors [53]. In the 1950s, Lloyd Old conducted a similar study using the Bacillus Calmette-Guérin (BCG) vaccine [54].

A new classification system for cancer vaccines has been proposed based on three key factors: the specific immunogenic antigen targeted in the tumor, the patients whose tumors express these antigens, and the method used to deliver these antigens to professional APCs. Vaccines are divided into two major categories: predefined vaccines, which target shared antigens common to multiple patients' tumors, and personalized vaccines, which are tailored to the unique antigens identified in each individual patient's tumor.

While recent progress in cancer vaccines has shown promise in shrinking tumors, extending survival, and offering renewed hope for patients [55–57], several challenges remain. Limitations such as small-scale trials, modest survival improvements, and significant resource demands have hindered wider implementation and created skepticism. These challenges are reminiscent of the early struggles faced by other successful cancer immunotherapies, such as monoclonal antibodies, which saw years of mixed results before the breakthrough of Rituximab in 1997 [58]. In a similar vein, anti-PD-1 therapies initially demonstrated no clear clinical benefit until the groundbreaking results achieved with Nivolumab [59]. Similarly, CAR-T cell therapy encountered numerous challenges over the years before achieving notable success [60]. Despite current limitations, the strong scientific foundation and encouraging preclinical data suggest that cancer vaccines may follow a similar trajectory to other successful immunotherapies, ultimately becoming standard cancer treatments. This review investigates evidence and proposes a definitive course of action, emphasizing vaccines as an essential instrument in the imminent battle against cancer. Additionally, cancer vaccines stimulate the production of new immune cells that specifically target the tumor, distinguishing them from checkpoint blockade therapies, which work by enhancing the activity of existing immune cells [61].

The popularity of more convenient therapies, such as enzalutamide, has diminished the excitement surrounding cancer vaccines, despite their modest survival benefits [62]. The approval of ipilimumab, a more convenient outpatient therapy with a stronger effect, overshadowed the survival benefits of a gp100 vaccine combined with intensive IL-2 treatment [63]. Similarly, a promising vaccine trial was eventually overshadowed by the introduction of a more manageable and effective chemotherapy regimen [64, 65].

The development of cancer vaccines has been the subject of extensive review in the literature, with a focus on the various forms of antigens used. These can range from whole tumor cells and tumor-derived proteins to peptides of different lengths, RNA or DNA (delivered directly or via viral vectors). Additionally, reviews have highlighted the adjuvants employed to enhance the immune response, such as carrier proteins, dendritic cells (DCs), CD40 ligand (CD40L), and chemicals like oil–water emulsions and Toll-like receptor (TLR) agonists [61].

A new classification system for cancer vaccines has been proposed based on three key factors: the specific immunogenic antigen targeted in the tumor, the patients whose tumors express these antigens, and the method used to deliver these antigens to professional antigen-presenting cells (APCs). Vaccines are divided into two major categories: predefined vaccines, which target shared antigens common to multiple patients' tumors, and personalized vaccines, which are tailored to the unique antigens identified in each individual patient’s tumor [61, 66].

Cancer vaccines generally target two main types of antigens. The first type is tumor-specific antigens (TSAs), which are unique to cancerous cells and arise from viral infections or mutations in the genes of the patient. Predefined personalized vaccines typically focus on TSAs, especially those caused by common genetic mutations linked to cancer that are shared among patients with similar HLA molecules [67]. The second category is tumor-associated antigens (TAAs), shared between cancer cells and normal tissues. While TAAs can be recognized by T cells, they often do not trigger a strong immune response because they are located within the cell. Most TAAs are found inside cancer cells, making them inaccessible to antibody-based therapies and T-cell therapies such as CAR-T cells. However, T cells can still recognize these intracellular TAAs through HLA molecules expressed on the tumor cell’s surface. To elicit a robust T-cell response, additional signals from APCs are required. Among APCs, DCs are especially important in “priming” T cells to attack cancer cells. A particular subset of DCs, known as cDC1s (type 1 conventional DCs), is particularly effective at “cross-presentation”. This process involves capturing antigens from outside the cell and displaying them on their surface for recognition by CD8+ T cells, enabling these T cells to target and destroy the cancer cells [55, 68, 69].

Cancer vaccines can enhance the efficacy of CAR-T cells by modulating the tumor microenvironment and increasing the expression of tumor-associated antigens. This dual approach not only improves the persistence and proliferation of CAR-T cells but also helps overcome challenges such as antigen escape and immune suppression within tumors. Recent research has explored innovative strategies for integrating cancer vaccines with CAR-T therapy, demonstrating significant advancements in tumor regression and long-term immune surveillance. These findings provide valuable insights into optimizing combinatorial immunotherapy approaches for more effective cancer treatment [70].

### Principles of cancer vaccination: immune activation and target antigens

Cancer vaccines efficiently load DCs with tumor antigens, triggering an immune response targeting various intracellular antigens. Different types differ in their delivery methods. A significant challenge in cancer vaccine development is the identification of the most efficacious antigen—the substance that can elicit a strong immune response. The ideal antigen should be tumor-specific, meaning it is exclusive to cancer cells to minimize the risk of damaging healthy tissue while also being highly immunogenic, sufficiently distinct from normal proteins to effectively activate T cells. Early vaccines primarily focused on TAAs such as gp100 or MUC1, which are present in both cancer cells and certain normal tissues. Although these antigens had broad applicability across patients, they often induced weak immune responses due to the body's pre-existing tolerance to these proteins [71, 72].

Cancer-testis antigens have emerged as a promising alternative. These antigens are typically expressed only during early development and are usually absent in healthy adult tissues. However, they can be reactivated in cancer cells, making them viable targets for immunotherapy. A well-known example of a cancer-testis antigen used in vaccine development is NY-ESO-1, which has been extensively studied in various vaccine frameworks [73].

Vaccines targeting mutated self-antigens have also shown promise. These mutations are present in the patient's genetic composition and are unique to their cancer cells. Examples of such mutations include the KRASG12D mutation and the ALK mutation in lung cancer [74, 75]. The advancement of genetic sequencing technologies has revealed a new class of potential targets: neo-antigens. These antigens arise from various genetic alterations in cancer cells, including mutations, insertions and deletions, gene fusions, and even the integration of viral or bacterial DNA [72, 76, 77].

Thus, choosing the right antigen is crucial for enhancing vaccine effectiveness while minimizing side effects. As research progresses, the range of potential targets expands, opening the door to the development of more personalized and powerful cancer vaccines. The emergence of next-generation sequencing has revolutionized cancer vaccine design, enabling the identification of neo-antigens unique to each patient. These antigens, arising from mutations specific to an individual’s tumor, offer the potential for highly personalized vaccine therapies. Biopsies from both tumor and healthy tissues of the same patient undergo comprehensive parallel sequencing to compare their DNA sequences. To identify mutations, advanced algorithms are used to compare the patient’s tumor and normal DNA to a reference genome, pinpointing mutations that are unique to the cancer cells [78]. Epitope prediction involves analyzing detected mutations using algorithms that assess their binding affinity to the patient's MHC molecules (major histocompatibility complex), which is essential for T cell recognition. Additionally, factors like antigen processing and abundance are considered in this evaluation [79]. The abundance of epitopes can be assessed through DNA or RNA sequencing to confirm whether the predicted antigen is present and actively expressed by the tumor cells [80, 81]. The most promising neo-antigens are then incorporated into a vaccine vector and delivered to the patient. Early studies in animal models have confirmed the potential of this approach. Researchers successfully identified tumor-specific peptides derived from mutations and used them to create therapeutic vaccines in mice [82]. Clinical trials have also shown promise. One study used exome sequencing and computational modeling to identify potential neo-antigens in melanoma patients. Vaccination with these neo-antigens resulted in the proliferation of T cells targeting tumor mutations, indicating that the vaccine may enhance the immune response against cancer [83]. Although the clinical significance of these initial trials is still being assessed, identifying neo-antigens paves the way for developing personalized cancer vaccines with the potential to significantly improve patient outcomes. The effectiveness of neo-antigen-based cancer vaccines relies heavily on the accuracy of epitope prediction algorithms for MHC-I and MHC-II molecules [79, 84, 85]. These algorithms are continually being refined to enhance their precision [86].

Historically, the emphasis has been on pinpointing neo-antigens with robust binding affinity to MHC molecules, based on the presumption that this would yield a more potent anti-tumor immune response [87]. Although this approach has achieved some success with MHC-I epitopes, predicting MHC-II binding remains challenging due to the greater structural flexibility of MHC-II molecules [84]. Advancements like the NetMHCIIpan algorithm are helping to address this challenge [88]. The use of artificial neural networks and deep learning offers promising opportunities to further enhance neo-antigen prediction [85].

A recent paradigm shift underscores the inadequacies of relying solely on tumor mutational burden (TMB) as a predictor of immunotherapy response [89–93]. Extensive studies indicate TMB may not be a reliable predictor when other influencing factors are considered [92]. Furthermore, the nature of mutations may be more critical, with persistent mutations in certain regions being more likely to correlate with a positive response to immunotherapy [93].

The key factor may not be the number of neo-antigens but their ability to trigger an immune response (immunogenicity) [92, 94]. Additionally, tumor heterogeneity adds another layer of complexity, as clonal TMB proves to be a stronger predictor of response compared to subclonal TMB [95]. Intriguing research by Jaeger et al., utilizing a genetically modified mouse model, indicates that simply increasing TMB through DNA mismatch repair deficiency (MMRd) is not enough to induce tumor immunogenicity. Notably, post-translational modifications, influenced by factors like HSP90 inhibition, also play a crucial role in shaping the tumor's “immunopeptidome”—the array of peptides displayed by MHC molecules [96].

These findings highlight the need for a deeper understanding of TMB evolution under immune pressure. Identifying the most suitable neo-antigens for immunotherapy requires a more advanced approach that goes beyond binding affinity to incorporate factors such as immunogenicity and the tumor's distinct immune environment.

### Types of cancer vaccines

#### Peptide-based approaches

**Fig. 3:**
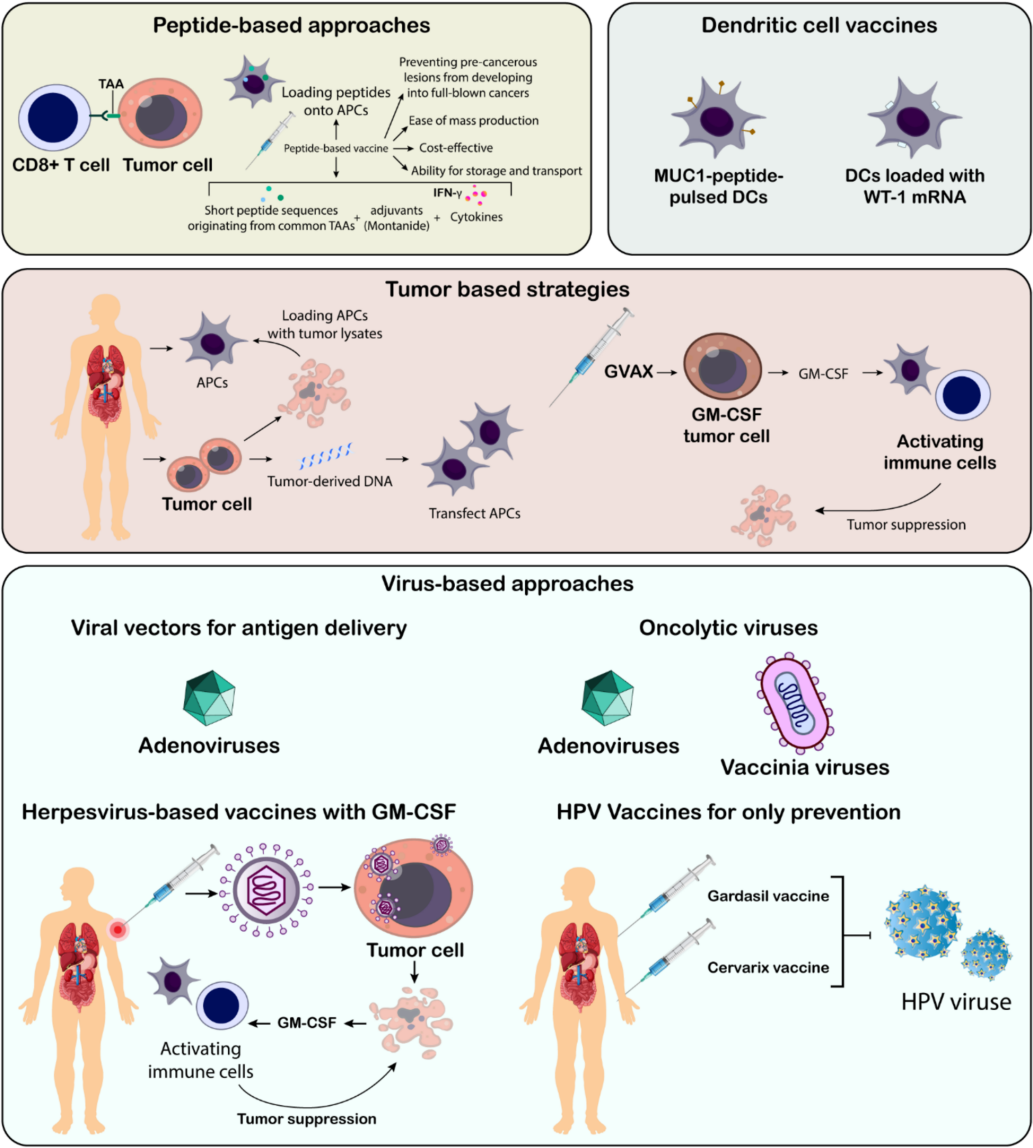
Cancer vaccines: different subtypes and platforms. An overview of various approaches in the development of cancer vaccines has been provided here, categorized into peptide-based, dendritic cell-based, tumor-based, and virus-based strategies. Peptide-based vaccines utilize short peptide sequences derived from tumor-associated antigens (TAAs) to stimulate CD8+ T cell responses. Dendritic cell vaccines involve loading antigen-presenting cells (APCs) with tumor-specific antigens, such as MUC1 peptides or WT-1 mRNA, to enhance immune activation. Tumor-based strategies include loading APCs with tumor lysates or transfecting them with tumor-derived DNA. Virus-based approaches encompass the use of viral vectors for antigen delivery (e.g., adenoviruses) and oncolytic viruses (e.g., adenoviruses, vaccinia viruses), as well as preventative vaccines against cancer-causing viruses like HPV (e.g., Gardasil, Cervarix). Each strategy aims to activate the immune system to recognize and eliminate tumor cells, ultimately leading to tumor suppression

Most cancer vaccines aim to activate a specific immune cell type, the CD8+ cytotoxic T cell, based on studies in mice that emphasize their pivotal role in combating cancer. Common strategies employed in these vaccines are outlined below (Fig. 3). Peptide-based vaccines are a widely used approach in cancer immunotherapy, designed to activate specific CD8+ T cells that target TAAs. Animal studies have demonstrated their considerable therapeutic potential [97–99]. Common TAA vaccines, combining short peptide sequences with adjuvants like Montanide and cytokines like GM-CSF or interferon-γ, show promising results in clinical trials [100–102]. Another approach involves loading peptides onto APCs, such as DCs, which has shown promising immune and clinical outcomes in smaller trials [103, 104]. Peptide vaccines are relatively cost-effective to produce due to their short length (around 9–10 amino acids) and ease of mass production. They are also stable for storage and transport. However, this approach is limited by a patient's HLA type, as the peptides must bind to specific HLA molecules. Patients who lack the targeted HLA types cannot benefit from this therapy. Additionally, traditional peptide vaccines primarily activate CD8+ cytotoxic T cells but fail to effectively stimulate CD4+ helper T cells, potentially weakening the overall immune response. To address this, non-tumor-specific "helper" peptides such as keyhole limpet hemocyanin (KLH) or PADRE peptides have been incorporated, although their exact mechanism of assistance remains unclear. Extended synthetic peptides (23–45 amino acids) have shown greater efficacy, likely due to improved processing and presentation, resulting in enhanced T cell activation [105–107]. Moreover, vaccines utilizing the full-length tumor antigen protein have not shown efficacy in phase III trials, even when combined with enhanced adjuvants. Administering multiple peptides targeting diverse T cell clones and antigens can offer advantages [108, 109]. Research indicates a correlation between enhanced survival and the diversity of the immune response, alongside a decrease in suppressive immune cells, including Tregs and MDSCs [109].

Peptide vaccines have shown potential in preventing the progression of pre-cancerous lesions into full-blown cancers, as evidenced by a recent trial targeting colon adenomas [110]. The study also highlighted the presence of immunosuppressive MDSCs in patients, suggesting their potential as a biomarker for vaccine response and indicating that earlier vaccination could yield better outcomes.

##### DC vaccines

Dendritic cells function as antigen-presenting cells that play a vital role in immune system regulation by taking up, processing, and displaying antigens for T lymphocyte recognition [111]. These cells are crucial for maintaining the balance between different T cell populations, specifically the CD4+ helper and CD8+ cytotoxic subsets. Multiple contemporary scientific reviews have thoroughly examined dendritic cell biology and their promising role in cancer treatment strategies through immunotherapy approaches [112, 113].

Due to the personalized nature of DC vaccines, clinical trials often involve individualized treatment plans and lack control groups. This complicates direct comparisons and makes it challenging to draw definitive conclusions about their overall effectiveness or the best approaches [114].

Researchers are exploring the use of various antigens in DC vaccines, including complex tumor lysates, synthetic MHC class I-restricted peptides, and mucin 1 peptide, a key antigen in cancer research [71, 115], WT-1 TAA mRNA is another highly ranked antigen [71, 116]. Different delivery methods, including intravenous, transdermal, and lymphatic injections, can impact clinical responses, which are influenced by various factors [114].

Preliminary research has validated the safety, feasibility, and immunogenicity of dendritic cell vaccines, with certain patients experiencing clinically significant tumor regression [114, 117, 118]. More recent trials (2004–2012) have shown encouraging results. For example, a study involving mucin 1 peptide-pulsed DCs combined with PADRE peptides and low-dose IL-2 therapy in kidney cancer patients demonstrated objective clinical and immunological responses [115]. Another trial with leukemia patients in remission showed improved outcomes following vaccination with DCs loaded with WT-1 mRNA [116]. These successful trials frequently combined DC vaccines with standard therapies and systemic cytokine treatment [119, 120].

#### Tumor based strategies

DC vaccines have potential in cancer immunotherapy, but further research is needed to improve their effectiveness. Early studies used modified tumor cells to elicit an immune response, with inactivated tumor cells producing immune-stimulating molecules like GM-CSF [121, 122]. This innovation led to the development of G-Vax, a vaccine platform based on genetically modified tumor cells engineered to express high levels of GM-CSF. G-Vax vaccines, which can be derived either from a patient’s own tumor (autologous) or from tumors of the same cancer type (allogeneic), have demonstrated potential in clinical trials, showing evidence of immune responses and even clinical improvement in some patients [123–125]. The preparation of personalized autologous tumor cell vaccines is complex and highly specialized. To expand beyond autologous approaches, researchers are exploring the use of established cancer cell lines in combination with the G-Vax platform. One such study involves pancreatic cancer patients undergoing a “prime-boost” regimen, starting with a vaccine using recombinant Listeria bacteria expressing the tumor antigen mesothelin, followed by a G-Vax vaccine derived from two allogeneic pancreatic cancer cell lines [126, 127]. This strategy offers the advantage of enabling multiple vaccinations without the risk of antibody-mediated suppression, while the use of bacteria mimics a natural infection, further enhancing immune system activation. Other personalized approaches utilize a patient’s tumor antigens, including ex vivo loading of APCs with tumor lysates or fusing tumor cells with APCs. These techniques have shown early promise in clinical trials, with some patients exhibiting immune responses to undefined components within the tumor lysates and even to foreign helper proteins incorporated in the vaccines [128, 129].

Researchers are investigating the utilization of tumor-derived DNA from a patient to transfect antigen-presenting cells, either sourced from the patient or established cell lines. This method allows the immune system to recognize and possibly attack mutated proteins specific to the patient’s cancer [130]. These strategies highlight the continued efforts to harness a patient's tumor as a source of antigens for cancer vaccines. Ongoing research focuses on optimizing these methods and determining the most effective ways to present tumor antigens to the immune system.

#### Virus-based approaches

As previously noted, incorporating pathogens into cancer vaccines can greatly amplify the immune response against tumor antigens. While peptide vaccines sometimes use TLR ligands (immune system activators) like CpG or polyIC, pathogens offer a complex array of molecules that stimulate multiple immune pathways [131–133].

##### HPV Vaccines for prevention only

The success of the Cervarix and Gardasil vaccines lies in their ability to prevent HPV-induced cervical cancer in uninfected adolescents. These vaccines activate humoral immunity against viral capsid proteins but are ineffective in treating existing cancers [131, 134].

##### Viral vectors for antigen delivery

Adenoviruses can function as vectors to deliver tumor antigens directly into easily transfected muscle tissue or introduce antigens into APCs ex vivo. Each virus uniquely affects APCs, with effects ranging from activation to suppression. A major challenge with direct viral vector administration is the potential for neutralizing antibodies that can hinder subsequent vaccinations, a concern less prominent in ex vivo approaches. Additionally, the clinical use of viral vectors presents complexities, including the need for “clinical-grade” virus production [131].

##### Prime-boost strategies with different viruses

A promising strategy employs a “prime-boost” approach using viruses with distinct backbones, each engineered to express tumor antigens. For instance, a prime-boost regimen for prostate cancer utilizes vaccinia virus and fowlpox virus, both expressing the TAA PSA along with costimulatory molecules. This method has shown improved patient survival and is currently undergoing further investigation [131]

##### Oncolytic viruses: a dual threat against cancer

Oncolytic viruses, including specific adenoviruses and vaccinia viruses, can selectively destroy cancer cells by replicating within them, controlled by tumor-specific promoters. Their selectivity can be further enhanced through engineered mutations in viral genes or the incorporation of chemokine genes [131, 135].

##### Herpesvirus-based vaccines with GM-CSF

Herpesviruses have shown potential as oncolytic vectors, particularly when engineered to include GM-CSF as an adjuvant or a growth factor for APC. T-VEC, a herpesvirus vector expressing GM-CSF, achieved a 26% objective response rate and an 11% complete response rate in a phase III melanoma trial. Additional research is required to elucidate the fundamental immune mechanisms that underpin this response [131, 136]. These viral strategies highlight the continued efforts to utilize viruses for delivering tumor antigens and activating the immune system to fight cancer. Ongoing research focuses on optimizing vector design, refining delivery methods, and exploring combinations with other therapies.

## Preclinical and clinical development of ani-leukemia vaccines

Cancer vaccines, a growing focus in cancer research, aim to harness the body’s immune system to fight cancer. These immunotherapies introduce tumor antigens into patients in various forms to stimulate the production of lymphocytes capable of targeting and destroying tumors. Researchers are actively developing cancer vaccines for various cancers, including B-cell leukemia, lymphoma, and cancers associated with specific mutations or viruses like Epstein-Barr virus (EBV). Although still under clinical investigation and not yet widely implemented, cancer vaccines hold significant potential to transform cancer treatment [137]. In the treatment of AML, two main vaccine strategies are distinguished by their design: peptide vaccines and DC-based vaccines. Both approaches aim to activate the immune system against AML cells through cellular and/or antibody-mediated responses. For an effective immune response and clinical benefit, the vaccine must target antigens that are highly expressed on AML blasts and, ideally, specific to these leukemia cells.

To assess the immune response, tetramer/pentamer staining is used to quantify the number of T cells activated by the vaccine by evaluating their binding affinity to complexes that mimic tumor antigens bound to MHC molecules. Two prevalent methods for cytokine detection are intracellular cytokine staining (ICS), which assesses cytokines synthesized within stimulated cells, and the enzyme-linked immunospot (ELISPOT) assay, which identifies cytokines upon their secretion by immune cells [137].

WT1, proteinase 3 (PR3), the receptor for hyaluronic acid-mediated motility (RHAMM), and mucin 1 protein (MUC1) are among the antigens being investigated for AML vaccines. These antigens have shown promise in eliciting cytotoxic T-cell responses against AML cells. WT1, a protein involved in cell growth regulation, has been linked to leukemia development. Research indicates that AML patients can mount immune responses against WT1, making it a viable target for vaccination. Phase I clinical trials have demonstrated the safety and tolerability of WT1 vaccines in AML patients. While early results suggest the potential to reduce relapse rates, particularly in high-risk individuals, larger trials are needed to validate these findings [138].

A small phase II trial involving 17 patients evaluated a WT1 peptide vaccine restricted by the HLA-A*0201 immune molecules in individuals with advanced AML. The vaccine was administered alongside granulocyte colony-stimulating factor (G-CSF). Forty-four percent of participants exhibited an immune response, marked by increased levels of WT1-specific T cells in the bloodstream. This response was particularly notable in patients with lower leukemia cell levels in the bone marrow. The research revealed no definitive correlation between immune response and clinical enhancement, with 10 patients attaining stable disease for a minimum of 8 weeks and one patient achieving complete remission. Interestingly, some patients, including the one who achieved complete remission, experienced a transient increase in leukemia cell counts, suggesting that a delay may be required for the immune response to take full effect. The small sample size limits definitive conclusions, particularly for patients with active disease, where the vaccine's efficacy may be less pronounced. These findings indicate that WT1 vaccines can elicit immune responses in AML patients, but further research with larger cohorts is essential to confirm their clinical benefits and refine their role in AML treatment. WT1 vaccine studies have indicated that higher levels of WT1 expression may correlate with stronger vaccine efficacy. This is because elevated WT1 expression often leads to a more robust immune response, as the vaccine targets WT1-expressing cells more effectively. However, variability in patient responses highlights the need for further research to confirm and refine these finding [139].

OCV-501, a WT1-derived vaccine targeting HLA class II molecules, was assessed in a phase II clinical trial involving 133 AML patients in remission who were ineligible for stem cell transplants. The trial compared the vaccine to a placebo but, unfortunately, did not detect any significant immune response in patients treated with OCV-501. Additionally, no differences were observed in disease-free survival or overall survival between the vaccine and placebo groups [140, 141]. The researchers suggested that limiting Class I epitopes to a specific HLA molecule (HLA-A*0201) might have restricted the CD8+ T cell response, as not all patients express this HLA molecule. This underscores a significant limitation of such approaches—HLA specificity may greatly reduce the pool of patients eligible for this type of vaccine therapy. These findings emphasize the need for further research to refine WT1 vaccine designs.

In a separate phase I/II trial, a vaccine designed to activate both CD8+ and CD4+ T cells was tested. This vaccine included two distinct epitopes restricted by the HLA-A2 molecule and a helper T cell epitope for HLA-DR (PADRE). It was administered to eight high-risk AML patients who were HLA-A*0201-positive and at varying stages of the disease. While immunological tests initially showed responses in 86% of patients, the study did not detect the formation of functional WT1-specific immunological memory. The vaccine demonstrated limited clinical efficacy, highlighting the critical importance of generating strong CTL memory for effective vaccination [142].

In CLL, evidence suggests the presence of multiple clones of leukemia-reactive CD4+ and CD8+ T cells in patients, indicating that a vaccine approach using whole tumor cells may be more effective than one targeting a single antigen. Whole tumor cell vaccines also reduce the likelihood of tumor escape variants that evade immune targeting. A critical step in developing DC-based immunotherapy for CLL is establishing reliable methods to stimulate T cells with leukemia antigens.

Initial studies compared different strategies for loading DCs with CLL antigens and assessed their ability to activate a patient’s T cells. One study evaluated DCs loaded with apoptotic CLL cells (Apo-DC) versus DC-tumor cell hybrids. A significantly higher proportion of DCs incorporated apoptotic bodies compared to those fused with tumor cells. Furthermore, only Apo-DCs induced a proliferative response in T cells from most patients (4 out of 5). While both methods triggered IFN-γ production in T cells, the response was notably stronger with Apo-DCs [143].

A study comparing Apo-DCs to tumor cell lysate or RNA demonstrated that they elicited a proliferative T-cell response, facilitated by MHC class I and II molecules. They also induced an increased quantity of T cells to produce IFN-γ, signifying a vigorous type 1 T cell response. Researchers emphasized the enhancement of Apo-DC production for clinical vaccine advancement [144, 145].

Researchers evaluated two distinct methodologies for isolating CD14+ precursor cells from CLL patients: immunomagnetic separation and counterflow elutriation. The investigation began with leukapheresis to obtain peripheral blood mononuclear cells (PBMCs) from eight individuals with CLL. The team employed counterflow elutriation for two patients' PBMCs, while the CliniMACS system (Miltenyi Biotec GmbH, Bergisch Gladbach, Germany) was utilized for immunomagnetic separation in the remaining six cases. These isolated monocytes were subsequently differentiated into dendritic cells (DCs) and exposed to apoptotic CLL cells to generate Apo-DCs. The immunomagnetic approach demonstrated greater efficiency, yielding more CD14+ cells with enhanced purity compared to elutriation, ultimately resulting in improved DC generation. Although cell loading capacity presented a limitation, utilizing multiple columns simultaneously (two or three) provided adequate monocyte precursors for most patients. Analysis revealed that 77% ± 4.3% of DCs successfully incorporated apoptotic material. Post-cryopreservation studies showed an 86% ± 4.4% recovery rate of Apo-DCs, maintaining 90% ± 2.8% viability. The Apo-DCs retained their characteristic phenotype, consistently expressing HLA-DR, CD80, CD83, and CD86 markers [143].

### Clinical trials of DC vaccines in hematological malignancies

#### Initial research on DC vaccines, as well as other alternatives in leukemias

Westermann and colleagues [146] studied unprimed DCs generated ex vivo in chronic myeloid leukemia (CML). Four infusions of DCs were administered in their cohort of ten CML patients, resulting in enhanced T-cell responses and cytogenetic/molecular improvements in four patients. In a phase II trial conducted by Cathcart et al. [147], patients with CML received immunization with a peptide derived from bcr/abl in combination with an immune adjuvant. Although most patients exhibited enhanced immune activity, only three experienced temporary cytogenetic improvements.

In addition to DC vaccines, peptide vaccines targeting leukemia-associated antigens, such as WT1, are also under investigation. Oka et al. [142] reported an immunological response in AML patients vaccinated with a WT1 peptide, which was linked to clinical improvements. Hobo et al. [148] described a phase I trial involving DC vaccines pulsed with mRNA encoding TAAs (MAGE3, Survivin, or BCMA) for myeloma patients; the vaccine was well-tolerated but produced minimal clinical responses. Van Tendeloo et al. [116] and Van Driessche et al. [149] achieved complete remission in AML patients by administering DC vaccines loaded with WT1 mRNA, which led to an increase in WT1-specific T cells.

Research into the use of apoptotic tumor cells to prime DCs is ongoing. Palma et al. [150] observed immune responses in CLL patients vaccinated with DCs loaded with apoptotic bodies, though no clinical responses were detected. Hus et al. [151] conducted trials using DCs primed with either whole tumor lysate or autologous tumor cells in patients with B-cell CLL, reporting clinical responses and an increase in leukemia-specific T cells in some cases. DiNicola et al. [152] documented both complete and partial responses in patients with relapsed non-Hodgkin's lymphoma treated with DCs loaded with heat-shocked and irradiated tumor cells (Table 1).

**Table 1:** Overview of clinical trials involving DC vaccines for leukemias

Type of leukemia	Research team	Intervention	Outcome	References
CML	Westermann et al	Unprimed ex-vivo DCs	Enhanced T cell responses, cytogenetic/molecular responses in 4 patients	[146]
CML	Cathcart et al	bcr/abl peptide + adjuvant	Increased immune activity, temporary cytogenetic improvement in 3 patients	[147]
CML	Litzow et al	Ex-vivo leukemic DCs	Increased CML-specific T cells, no clinical responses	[163]
AML	Oka et al	WT1 peptide vaccine	Immunological response, clinical improvements	[38]
AML	Van Tendeloo et al	DCs loaded with WT1 mRNA	Complete remission in some patients, increased WT1-specific T cells	[116]
AML (remission)	Roddie et al	DCs derived from leukemia	Immune responses, limited clinical benefit (2/5 remained in remission)	[164]
AML/MDS (post-HSCT)	Ho et al	Irradiated, GM-CSF-secreting autologous tumor cells	Good tolerability, potential NK cell activity benefit	[154]
CLL	Palma et al	DCs loaded with apoptotic bodies	Immune responses, no clinical responses	[150]
CLL	Hus et al	DCs with whole tumor lysate/autologous tumor cells	Clinical responses, increased leukemia-specific T cells in some patients	[151]
NHL	DiNicola et al	DCs loaded with heat-shocked/irradiated tumor cells	Complete & partial responses	[152]
Myeloma	Hobo et al	DCs pulsed with mRNA-encoded antigens	Well-tolerated, minimal clinical responses	[148]

#### DC/AML fusion cell vaccines in remission

A recent clinical trial was launched to evaluate DC/AML fusion cell vaccines in AML patients in remission. Initial findings suggest promising remission rates [153]. A novel approach involves immunizing patients with irradiated autologous tumor cells engineered to secrete GM-CSF, aiming to enhance the maturation and function of DCs directly in vivo [154, 155]. Ho and colleagues observed high tolerability and potential benefits for NK cell activity in high-risk AML/MDS patients after undergoing allogeneic HSCT [154].

One strategy to overcome deficiencies in DCs is to generate mature DCs directly from the immature myeloid cells of leukemia patients, as demonstrated in CML [156, 157] and AML [158–160], eliminating the need for separate antigen loading. Alternatively, leukemia blast cell lines can be induced to differentiate into DCs capable of presenting antigens [161], potentially eliciting an immune response against a broader range of leukemic antigens. However, re-infusing DCs derived from leukemia raises concerns regarding potential immune escape mechanisms [162].

Litzow and colleagues reported an increase in CML-specific T cells in CML patients vaccinated with leukemic DCs generated ex vivo, some of whom were receiving Imatinib; however, no clinical benefits were observed [163]. Roddie et al. immunized AML patients in remission with leukemia-derived cells resembling DCs, noting immunological responses but limited clinical outcomes, as only 2 out of 5 patients remained in remission. One patient developed an autoimmune-related rash [164]. A notable limitation is that not all patients can generate leukemia-derived DCs, often due to mutations or the absence of CD14 expression [165, 166]. The immunosuppressive tumor microenvironment [167] in advanced leukemia may limit the effectiveness of DC vaccines [168, 169]. Immune responses may be diminished by factors including regulatory T cells, myeloid-derived suppressor cells, and soluble inhibitory molecules. Future research on vaccination strategies post-chemotherapy and transplantation shows promise for eliminating minimal residual disease and preventing relapse. Our results indicate that the post-allograft phase may represent an optimal opportunity for immunotherapy. During this period, the immune system experiences lymphopoietic reconstitution, and the presence of immunosuppressive regulatory T cells, which may impede the vaccination response, is diminished [170].

A promising approach to enhance vaccine performance involves combining vaccines with immune-enhancing substances. Researchers have explored using cytokines like IL-2 alongside various immunotherapeutic treatments in preclinical investigations [171]. In clinical studies utilizing DCs engineered to release specific compounds, GM-CSF has been a primary focus. Borello and colleagues conducted a phase II AML study where patients received induction chemotherapy followed by immunotherapy using genetically altered K562 tumor cells designed to produce elevated GM-CSF levels [172]. After autologous stem cell transplantation, patients' lymphocytes were harvested and reinfused. This immunotherapeutic intervention decreased WT1 transcripts, an indicator of leukemia cells, in 69% of participants after initial dosing and boosted immune cell functionality. Patients receiving immunotherapy demonstrated significantly higher overall survival compared to those who couldn’t receive it (73.4% versus 57.4%). Nevertheless, only a small subset of patients showed measurable GM-CSF levels, and the research wasn't structured to definitively contrast modified K562 cells with their unmodified versions. The myeloma treatment lenalidomide possesses immunomodulatory characteristics that remain under investigation but are believed to enhance T cell and NK cell functionality. Laboratory research indicates that lenalidomide, especially when used with pomalidomide, may influence DCs, potentially strengthening their antigen presentation capabilities [173]. Furthermore, preclinical evidence suggests lenalidomide can amplify vaccination immune responses [174], prompting a planned clinical trial combining these approaches. The PD-1/PD-L1 pathway serves as a critical regulator of T cell activation and anti-cancer targeting ability. Cancer patients often exhibit increased PD-1 expression on T cells that interacts with PD-L1 on malignant cells, diminishing T cells’ cancer-fighting capacity [170, 175]. Following autologous stem cell transplantation for myeloma, T cell PD-1 expression normalized. Our research examined how PD-1 blockade affects T cell responses to DC/tumor cell fusions. Administration of the anti-PD-1 antibody (CT-011) redirected T cell responses toward a more activated Th1 profile and reduced regulatory T cell populations, resulting in improved tumor cell killing in laboratory testing. A current clinical trial is assessing the combination of PD-L1 blockade with DC/tumor fusion cell vaccination in AML patients [170].

## CAR-T cell therapy: mechanistic insights

### CARs’ structure and nature

Chimeric antigen receptor (CAR) T cell therapy exemplifies a revolutionary advancement in cancer therapeutics, utilizing engineered T lymphocytes to specifically target malignant cells. This therapeutic approach relies on meticulously engineered receptors comprising multiple functional elements that work in concert to guide T cells toward cancer-specific targets. The key structural components include the antigen-recognition domain, spacer region, membrane-spanning segment, and cytoplasmic signaling modules, each contributing distinctly to therapeutic efficacy [176].

The antigen-recognition component typically consists of antibody-derived variable heavy (VH) and light (VL) chains, combined into a single-chain fragment (scFv). This structure enables direct recognition of tumor surface antigens without requiring major histocompatibility complex (MHC) presentation. Notably, some CAR designs have been developed to recognize intracellular tumor antigens through MHC-dependent mechanisms, mimicking natural T cell receptor (TCR) function [177, 178].

Between the antigen-recognition and membrane-spanning segments lies the spacer or hinge region, which provides crucial flexibility for optimal antigen engagement. The design specifications of this region vary according to the target antigen's characteristics and location on the tumor cell. Shorter spacers have shown better efficacy for targets like CD19 and carcinoembryonic antigen (CEA), while longer spacers are preferred for mucins and membrane-proximal ROR1 epitopes [176].

The composition of the spacer, commonly derived from CD8, CD28, or immunoglobulins, significantly impacts functionality. However, immunoglobulin-based spacers may interact with Fcγ receptors, potentially compromising CAR-T cell persistence [179–183].

The membrane-spanning domain, though historically less studied, has emerged as a critical determinant of CAR functionality [184, 185]. This component, typically derived from proteins such as CD3ζ, CD4, CD8α, or CD28, serves beyond mere membrane anchoring. The CD3ζ-derived transmembrane domain enhances T cell activation through CAR dimerization and TCR complex integration, despite showing lower stability compared to CD28-derived alternatives [184, 186].

Research has demonstrated that the combination of transmembrane and spacer components influences cytokine production patterns and cellular survival. For example, constructs incorporating CD8α-derived elements exhibit reduced TNF and IFNγ production and greater resistance to activation-induced cell death compared to CD28-derived counterparts [187]. These observations suggest that effective CAR-T signaling requires compatible transmembrane and intracellular signaling domains. However, for superior CAR expression and stability, the widely used CD8α or CD28 transmembrane domains may be preferable. As research advances our understanding of transmembrane domains, scientists can refine CAR designs for improved functionality [176]. CAR-T cell therapy has evolved considerably since its inception, with significant focus on understanding co-stimulation's role in these engineered cells. A key aspect of this development is the progression of CAR co-stimulation strategies. Developed in the late 1990s, first-generation CARs relied solely on CD3ζ or FcRγ signaling domains for activation but achieved only modest results [60]. Although they triggered some T cell activation via CD3ζ motifs, these signals proved insufficient for generating strong and persistent T cell responses [188, 189]. This ineffectiveness was reflected in poor clinical outcomes with minimal observed efficacy [190, 191]. In the clinically successful second generation, scientists recognized co-stimulation's essential role and incorporated a co-stimulatory domain alongside CD3ζ [192, 193]. The frequently used co-stimulatory domains, CD28 and 4-1BB (CD137), have demonstrated effectiveness in clinical trials, resulting in better patient response rates [194]. CD28-based CAR-T cells develop into effector memory cells that primarily use aerobic glycolysis for energy, while 4-1BB-based CAR-T cells mature into central memory cells with enhanced mitochondrial activity [194]. Second-generation CAR-T cells have shown notable efficacy in treating various blood cancers, including CLL, ALL, and MM [195]. Their potential effectiveness against solid tumors remains under active investigation. In early third-generation development, researchers hypothesized that a single co-stimulatory domain might be inadequate. Consequently, they created third-generation CARs combining two co-stimulatory domains with CD3ζ [196]. Preclinical results for these designs have been mixed. Some studies report increased cytokine production and improved antitumor responses in lymphoma models [197], while others show no significant advantage over second-generation CARs in leukemia and pancreatic cancer models [198, 199]. Research continues on additional co-stimulatory domains such as ICOS, CD27, and OX40, though these remain in the preclinical phase [200–202]. With increasing insights into how co-stimulation affects CAR-T cell functionality, future CAR designs can be further optimized to enhance their effectiveness in cancer therapy.

### CAR engineering and manufacturing

**Fig. 4:**
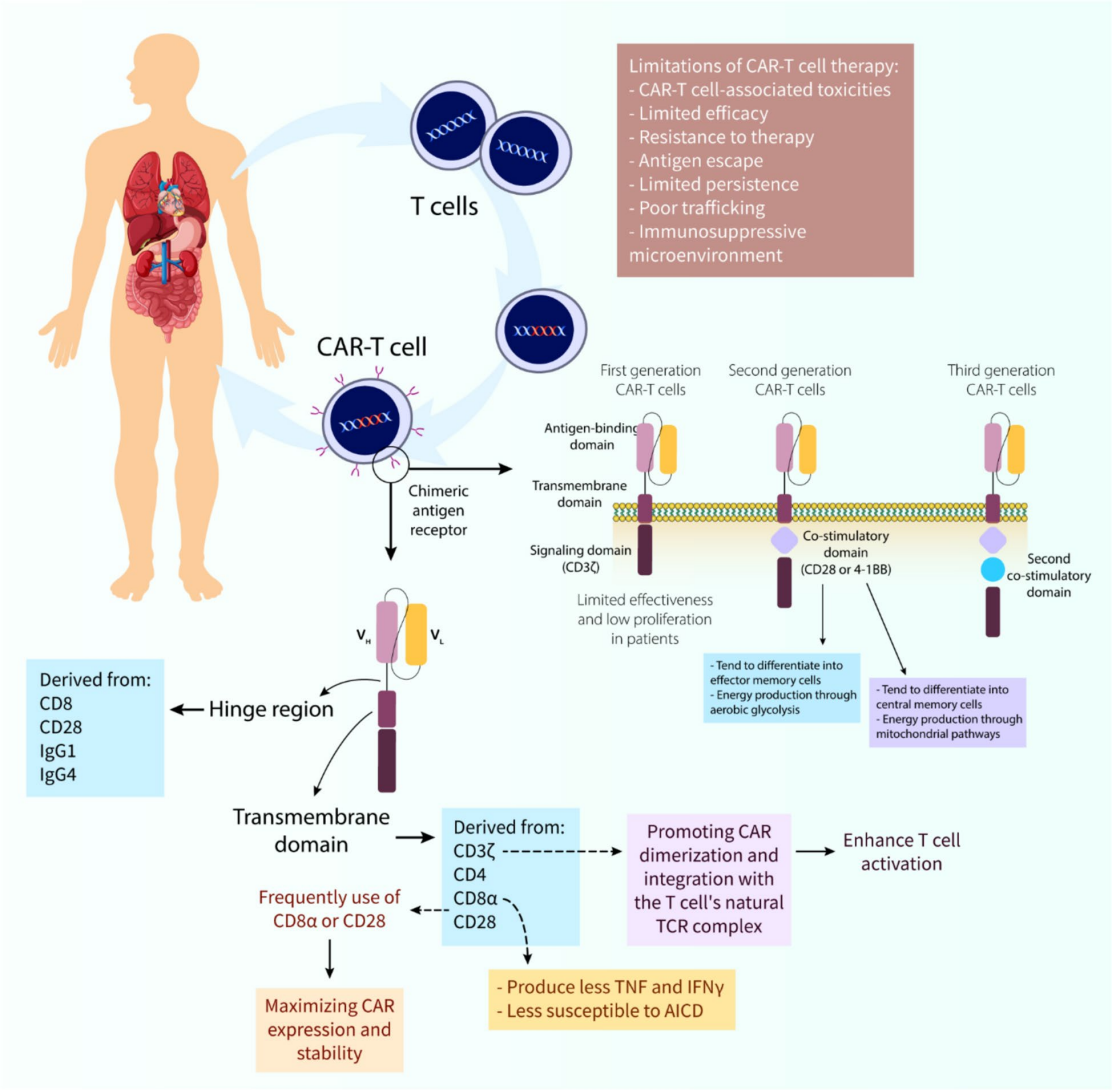
CAR-T cells and CAR-T cell therapy: structure, mechanistic insights, and engineering. The fundamental principles of CAR-T-cell therapy highlight its mechanism of action where engineered T cells expressing a CAR-Target tumor-specific antigens (e.g., CD30). The diagram outlines the structural components of a CAR, including the antigen-binding domain, hinge region, transmembrane domain, and intracellular signaling domain (CD3ζ). It further depicts the evolution of CAR design from first to third generations, emphasizing the incorporation of co-stimulatory domains (e.g., CD28, 4-1BB) to enhance T-cell activation, proliferation, and persistence, shifting the T-cell phenotype towards central memory cells with mitochondrial-based energy production. Key limitations of current CAR-T cell therapies include associated toxicities, limited efficacy, resistance mechanisms, antigen escape, poor persistence and trafficking, and the influence of the immunosuppressive tumor microenvironment

The emergence of CAR-T cell therapy marks a significant advancement in cancer therapeutics, particularly in treating blood-related cancers (Fig. 4). Initial CAR designs incorporated antibody-derived antigen recognition elements combined with TCR signaling components and a transmembrane segment [203–207]. However, these first-generation constructs demonstrated limited durability and expansion capabilities in clinical applications, leading to poor therapeutic outcomes [208]. A significant breakthrough occurred with the development of second-generation CARs, which incorporated additional co-stimulatory elements such as CD28 or 4-1BB [209, 210]. These modifications substantially enhanced T cell activation and longevity. The remarkable efficacy of CD19-targeting second-generation CAR-T cells against B-cell leukemia [211–213] ultimately led to FDA approval for specific cancer indications in 2017. The manufacturing of CAR-T cells exemplifies individualized medicine, beginning with the harvesting of a patient’s own T lymphocytes (autologous approach) to prevent immunological rejection [214, 215]. These cells undergo activation through TCR complex stimulation coupled with co-stimulatory signals, typically involving CD28 [216]. This activation process utilizes various methodologies, including antibodies, specialized beads, or engineered antigen-presenting cells [216, 217]; while specific cytokines like IL-2 help shape the cellular phenotype [218–220]. The genetic modification process involves introducing the CAR construct using either viral or non-viral delivery systems [221, 222]. Following genetic modification, the engineered cells undergo expansion in specialized bioreactors before patient reinfusion [223]. Unlike conventional cancer treatments, CAR-T therapy typically involves a single administration or split-dose protocol. The field continues to advance, with current research exploring advanced gene-editing platforms such as CRISPR to enhance both the safety profile and therapeutic efficacy of CAR-T cells [224, 225].

Recent findings have expanded the use of CAR-T cell therapy beyond blood cancers. A 2024 publication highlighted ongoing research into using CAR-T cells to treat autoimmune conditions like myasthenia gravis and multiple sclerosis, where standard treatments have fallen short [226]. This exploration reflects a move toward broader uses of CAR-Technology beyond just cancer treatment. Furthermore, improvements in manufacturing processes and next-generation CAR constructs are intended to increase their therapeutic potential. A recent review highlighted innovative strategies designed to overcome the challenges posed by solid tumors, which have historically limited CAR-T cell efficacy [227]. These approaches involve refining antigen targeting, increasing their durability within the TME, and developing allogeneic CAR-T cells that can serve as readily available, off-the-shelf treatments [228].

As research continues to advance, we can look forward to even greater improvements in this promising cancer therapy. The expanding exploration of CAR-T cell applications across different diseases underscores its potential to reshape treatment approaches not only in oncology but also in other complex medical conditions. Through ongoing innovation and collaboration across.

Research and clinical domains, CAR-T cell therapy holds the promise of better patient outcomes and a transformative impact on healthcare.

### Advancements in CAR-T cell engineering: improvements for effectiveness

#### Gene transfer

**Fig. 5:**
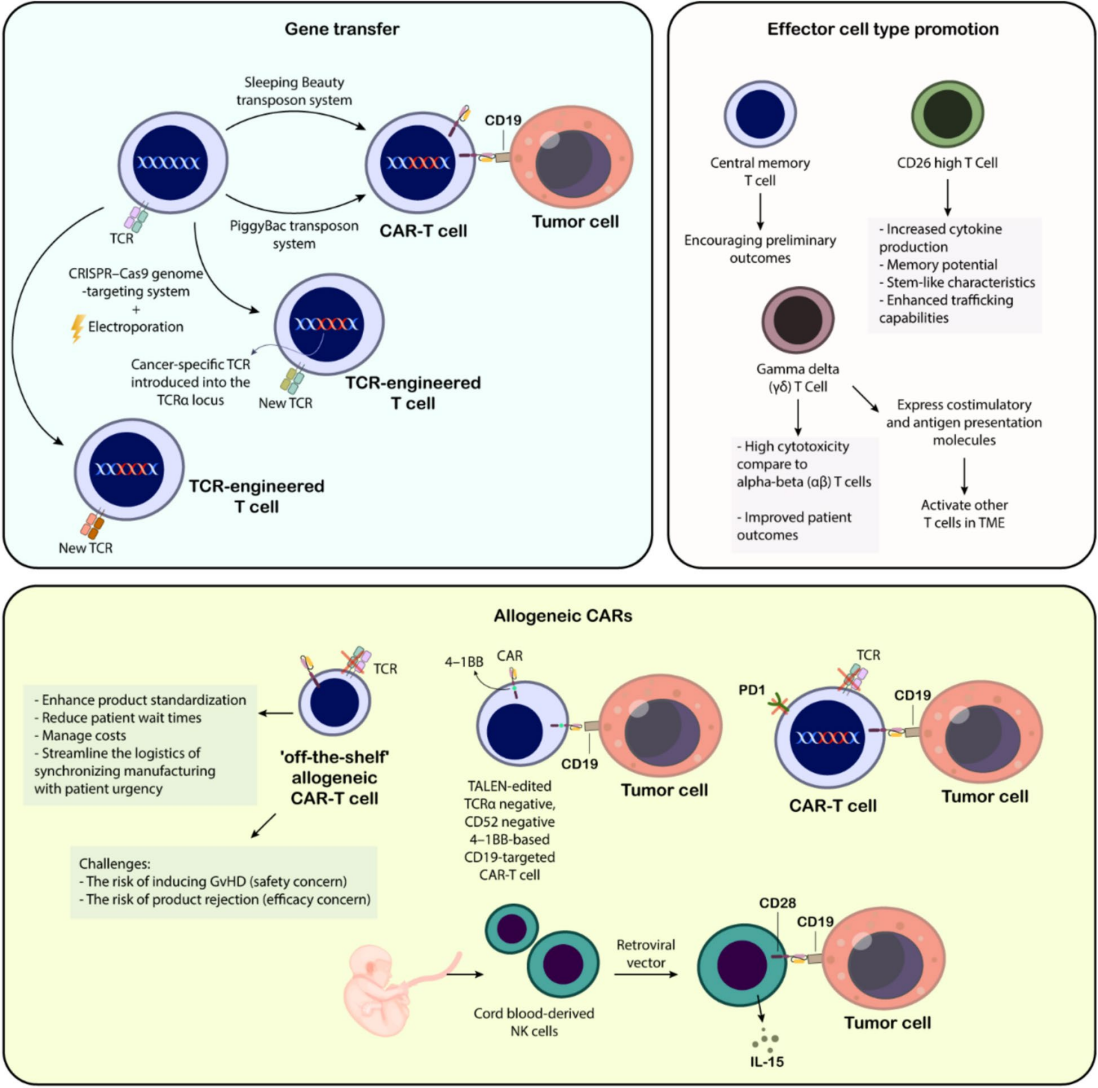
Advancements in adoptive cell therapy for cancer: gene transfer technologies, effector cell type promotion, and allogeneic approaches. The top left section illustrates various gene transfer technologies, including viral vectors (retroviral, lentiviral—implied), non-viral methods like electroporation, and transposon systems (Sleeping Beauty, PiggyBac) used to engineer T cells with CARs or cancer-specific T-cell receptors (TCRs). CRISPR-Cas9 genome editing for targeted TCR replacement is also depicted. The top right section focuses on effector cell type promotion, highlighting the benefits of generating central memory T cells and CD26 high T cells, which exhibit enhanced cytokine production, memory potential, stem-like characteristics, and trafficking capabilities. Additionally, the role of gamma delta (γδ) T cells, known for their high cytotoxicity and ability to express costimulatory and antigen presentation molecules, as well as activate other T cells within the tumor microenvironment (TME), is presented. The bottom section details allogeneic CAR-T cell therapy, emphasizing its potential to enhance product standardization, reduce patient wait times, manage costs, and streamline logistics for “off-the-shelf” availability. Different strategies for allogeneic CAR-T cells are shown, including TALEN-edited TCRα and CD52 negative CAR-T cells targeting CD19 with a 4-1BB costimulatory domain, and CAR-T cells with engineered PD1 disruption. The use of cord blood-derived NK cells engineered with CARs via retroviral vectors and stimulated with IL-15 is also illustrated as an alternative allogeneic ACT approach. The challenges associated with allogeneic CAR-T therapy, namely the risk of GvHD and product rejection, are also noted. This figure collectively showcases the multifaceted efforts to improve the efficacy, safety, and accessibility of adoptive cell therapies for cancer

A key obstacle facing genetically engineered T-cell therapies is their dependence on viral vectors, which are both expensive and time-consuming to produce for clinical applications [203]. The DNA capacity of viral vectors is limited, with adeno-associated viruses having a limit of 4 kb, adenoviruses at 8.5 kb, and lentiviral vectors at 10 kb. Transposons have been explored as a cost-effective, non-viral alternative for introducing genes into CAR-T cells. CD19-targeted CAR-T cells, generated using the Sleeping Beauty transposon system, have been used to treat leukemia and lymphoma patients who relapsed after allogeneic HSC transplants. The piggyBac transposon platform is also a promising approach for CAR-T cell production, with a biotechnology company using it in two clinical trials [229]. Roth and colleagues developed a non-viral method to deliver DNA fragments larger than 1 kb to human T cell sites using CRISPR–Cas9 and double-stranded DNA electroporation. This integrated a cancer-specific TCR into the TCRα locus, resulting in robust anti-tumor responses in vitro and in vivo [230]. To avoid unwanted pairing between the endogenous and introduced TCR chains, which can occur during standard transgenic TCR production, the approach involves simultaneously orthotopically replacing the endogenous TCRα and β loci. This ensures that the engineered T cells closely mimic normal T-cell function [231]. This approach faces clinical challenges, particularly for scenarios involving large genetic cargo like extensive CAR constructs. Beyond adjustments in genetic information transfer, ramping up CAR-T cell production requires ex vivo T cell expansion. To improve this process, researchers have introduced mimic cytokines, potentially enhancing manufacturing efficiency. Historically, various groups have sought the ideal cytokine combinations to foster optimal T-cell development conditions (Fig. 5) [232].

#### Effector cell type promotion

Currently, CAR-T cell therapy draws upon various T cell types sourced from the patient, but researchers are exploring ways to improve its effectiveness. Certain T cell subsets may hold greater therapeutic potential than others. Central memory T cells have been utilized to create CD19-targeted CAR-T cells, with promising early results. The persistence of CAR-T cells appears to be more closely linked to the chosen co-stimulatory domain than the specific T cell memory subset. Additionally, T cells with high CD26 expression show promise in solid tumor models, demonstrating enhanced cytokine production, memory capacity, stem-like traits, and improved trafficking abilities [233]. Moreover, gamma delta (γδ) T cells offer distinct advantages. Their cytotoxicity is on par with the alpha–beta (αβ) T cells commonly used in CAR-T cell therapy, yet they experience less exhaustion and exhibit lower levels of inhibitory markers such as TIM-3 and PD-1 [234, 235]. Moreover, γδ CAR-T cells exhibit costimulatory and antigen presentation molecules, which may allow them to activate other T cells within the TME [235]. Notably, the presence of γδ T cells within tumors has been linked to better patient outcomes. γδ T cells are indeed promising for cancer immunotherapy due to their ability to target tumors in an MHC-independent manner. They show strong cytotoxicity against a wide range of malignancies, including hematologic cancers, and have demonstrated potential in enhancing immune responses when combined with other therapies. However, it is important to emphasize that clinical data on γδ T cells remain relatively sparse. Most studies are still in preclinical stages, focusing on optimizing expansion methods, improving tumor specificity, and overcoming challenges related to their persistence and efficacy in vivo [236].

Researchers are developing a split, universal, and programmable (SUPRA) CAR system to develop universally applicable CAR-T cells for diverse patients and tumor types. This system uses a single vector to encode a “universal” CAR with a “zipper” mechanism, allowing for precise targeting of specific antigens and fine-tuning of the T-cell response to different protein therapeutics [237]. This approach could potentially be combined with a second zipper system incorporating a costimulatory domain. Other strategies, such as biotin-binding and switch module CARs, utilize CARs engineered to recognize specific tags like biotin or FITC. In these systems, an antibody therapy targeting the tumor antigen is conjugated to the corresponding tag, enabling CAR-T cells to detect and eliminate the tagged tumor cells [238–240]. These advancements aim to create more potent and adaptable CAR-T cell therapies, paving the way for a more universal approach to this promising cancer treatment (Fig. 5).

Third-party allogeneic CAR-T cell therapies aim to enhance product consistency, reduce patient wait times, streamline urgent patient needs coordination, and control costs [241]. Allogeneic T-cell therapies face challenges such as GVHD risk, safety concerns, and product rejection. In 2017, the first clinical use of gene-edited T cells was used to treat two infants with B-cell ALL. Both patients achieved full recovery with minimal GVHD and underwent allogeneic stem cell transplantation, the standard treatment for remission [242]. Phase I/II clinical trials investigating this CAR in pediatric and adult ALL are underway (NCT02746952193, NCT02808442194).

CRISPR-Cas9 technology is being used for multiplex genome editing in allogeneic CAR-T cell products, aiming to reduce risks of GVHD, rejection, and T-cell exhaustion by targeting endogenous TCR, β2 microglobulin, and PD1 loci through lentiviral delivery [243]. More recently, Liu et al. [244] utilized NK cells derived from cord blood, transducing them with a CD28-based CD19-targeted CAR using a retroviral vector. Cord blood offers a distinct advantage due to its higher proportion of NK cells (30% of lymphocytes) than peripheral blood (10%), significantly reducing the risk of T cell contamination and the associated GVHD. To improve NK cell persistence, the researchers incorporated an IL-15 transgene into the construct. Their initial clinical trial showed promising responses and prolonged remissions in lymphoma patients, and both therapies are now advancing through clinical development [245]. Daher et al. [246] utilized CAR NK cells derived from cord blood with a CISH deletion to target the cytokine-inducible SH2-containing protein, which suppresses IL-15 signaling. This modification enhanced the IL-15 design, resulting in CARs with improved efficacy in eliminating lymphoma xenografts in vivo and increased aerobic glycolysis (Fig. 5).

## Clinical application of CAR-T cell therapy in leukemias

The therapeutic landscape for leukemia treatments has been transformed by CAR-T cell therapy innovations (Table 2). Among various targets, CD19 has emerged as the most significant for CAR-T cell applications, given its abundant expression in B cell cancers while being minimally present in healthy B cells. Early experimental research in the United States showed promising results for B cell ALL treatment. At Memorial Sloan Kettering Cancer Center (MSKCC), researchers achieved significant breakthroughs by successfully treating aggressive B-ALL in experimental mouse models, utilizing a modified second-generation CD19 CAR incorporating CD3ζ signaling and CD28 co-stimulation mechanisms [247]. Subsequently, researchers at the University of Pennsylvania made further advances by investigating different co-stimulatory components, notably identifying that incorporating the 4-1BB (CD137) domain led to improved T cell functionality and survival rates. These pioneering studies catalyzed numerous clinical investigations examining various aspects of CAR design, T-cell manufacturing protocols, gene transfer methods, and treatment regimens, consistently demonstrating efficacy in CD19-directed CAR-T therapy for B-ALL patients [248].

**Table 2:** CAR-T cell therapies for leukemias

CAR-T design	Disease target	Key findings	References
CD19 CAR with CD3ζ signaling and CD28 co-stimulation	B-ALL	Successfully eliminated aggressive B-ALL in murine models	[247]
CD19 CAR with 4-1BB (CD137) co-stimulatory domain	B cell malignancies	Enhanced T cell activity and persistence compared to other domains	[199]
19–28z CAR-T cells	Relapsed B-ALL (adults)	All 5 patients achieved MRD-negative complete remission; CAR-T cells persisted 3–8 weeks	[214]
19–28z CAR-T cells	Relapsed B-ALL (expanded cohort)	14 of 16 patients achieved complete remission; no GVHD in post-transplant patients	[249]
CD19 CAR-T cells	Pediatric B-ALL	14 of 20 patients achieved complete remission; CAR-T cells detectable up to day 68	[250]
UPenn/CHOP	CTL019 (CD19 CAR with 4-1BB co-stimulation, lentiviral vector)	B-ALL	[252, 253]
CD19 CAR-T cells	CLL	CAR-T expansion correlated with positive tumor responses; steroids for CRS may limit persistence	[211]
Anti-CD33 and anti-CD123 CAR-T cells	AML	CAR-T cells eradicated AML but also destroyed normal hematopoietic cells	[260]

In clinical investigations at MSKCC, researchers evaluated CD19 CAR-T cell treatment in adult patients with recurrent B-ALL who had not previously received allogeneic stem cell transplants. Five patients were administered second-generation 19-28z CAR-T cells [214]. The participants exhibited varying degrees of disease progression, from minimal residual disease in remission to severe cases with 70% bone marrow blast involvement. Following CAR-T cell administration, all participants achieved complete remission with undetectable minimal residual disease. Subsequently, four patients underwent stem cell transplantation, which affected the ability to gather extended data on CAR-T cell persistence. Prior to transplantation, B cell recovery was observed in all patients, indicating diminished CAR-T cell activity or depletion. Post-infusion monitoring revealed CAR-T cells remained detectable in blood and bone marrow samples for 3–8 weeks. The sole patient unsuitable for transplantation experienced disease recurrence at day 90, potentially due to steroid treatment for side effects that may have compromised CAR-T cell persistence. The research was later expanded to include 11 more patients with relapsed B-ALL, bringing the total number of treated subjects to 16 [249]. These results highlight CD19 CAR-T therapy's potential effectiveness for relapsed B-ALL, though further investigation is needed regarding long-term persistence and treatment optimization. A follow-up MSKCC study focused on four patients with previous allogeneic stem cell transplantation [249]. This research showed that 14 of 16 patients receiving 19–28z CAR-T cells achieved complete remission, with exceptions being one patient with extensive bone marrow infiltration and another with extramedullary disease. Following CAR-T therapy, two patients were deemed transplant-ineligible, two declined the procedure, and one remained under evaluation. Of the 16 patients, seven underwent stem cell transplantation, with two fatalities from transplant-related complications. Though complete long-term data wasn't available for all participants, no relapses occurred among transplant recipients (follow-up ranging 2–24 months). Notably, none of the four patients with previous stem cell transplantation developed GVHD after CAR-T therapy [250].

A Phase I clinical investigation was implemented by the National Cancer Institute (NCI) to assess CD19 CAR-T treatment efficacy in young patients with B-ALL [250]. Study participation was open to patients with detectable B-ALL, including those exhibiting elevated leukocyte counts. Patients who had passed the 100-day post-transplant milestone without developing GVHD were considered eligible for allogeneic stem cell transplantation, though cases involving central nervous system disease were excluded. The study population included six B-ALL patients whose disease had proven resistant to initial treatments. Within the cohort, eight participants had previously undergone allogeneic stem cell transplantation, and one had received unsuccessful CD19 CAR-T treatment at another facility. Through dose-escalation methodology, researchers determined the optimal tolerated dose to be 1 × 10^6^ cells/kg. The treatment yielded complete remission in fourteen out of twenty participants. Among these, ten patients proceeded to receive allogeneic stem cell transplantation and maintained their remission status. Two patients who achieved MRD-negative complete remission were unable to proceed with transplantation due to health complications and subsequently experienced disease recurrence with CD19-negative leukemia. Additional T cell infusions were administered to three non-responding patients, but these interventions proved unsuccessful. CAR-T cells became undetectable in all participants by day 68, with ten patients undergoing stem cell transplantation between 45–82 days following the initial infusion. Aligning with MSKCC’s observations, patients who received CAR-T therapy following allogeneic stem cell transplantation showed no signs of GVHD [251].

Research teams from the University of Pennsylvania and Children's Hospital of Philadelphia initiated clinical trials investigating CTL019, an enhanced version of CD19 CAR-T therapy, for treating B-ALL patients [252, 253]. CTL019 differs from MSKCC and NCI designs by incorporating 4-1BB co-stimulation instead of CD28 and using a lentiviral rather than retroviral vector. The first patient, a child with relapsed B-ALL, received high-dose CTL019 cells and experienced severe cytokine release syndrome (CRS), prompting dose reduction for safety. Both the first patient (treated over 9 years ago) and the second (with previous cord blood transplant) achieved complete remission by day 28 post-infusion. However, the second patient relapsed with CD19-negative leukemia 2 months later. In the expanded study with 2-year follow-up, thirty ALL patients (25 pediatric, 5 adult) received CTL019 cells [254, 255].

Various scientific organizations have explored CD19 CAR-T therapy for relapsed or high-risk CLL patients [211, 213, 256, 257]. Early studies showed substantial CAR-T expansion after infusion correlating with positive tumor responses. However, steroid use for managing CRS may limit CAR-T persistence and reduce effectiveness [211].

University of Pennsylvania researchers investigated long-term outcomes of the first 14 CLL patients treated with CTL019 [257]. Patients had undergone median five previous treatments with median age 67 years. The overall response rate reached 57%, with four complete and four partial remissions. Similar to ALL findings, durable remissions correlated with CAR-T expansion and persistence. All complete remission patients were MRD-negative. Remarkably, two patients maintained remission over 5 years post-infusion with detectable CAR-T cells [257]. While CLL response rates are promising, they fall below the 70–90% complete remission rates in ALL. This difference may result from functional T cell impairments in CLL patients, CAR-T product variations, and CLL microenvironment differences affecting CAR-T effectiveness. Currently, no clinical characteristics reliably distinguish responders from non-responders in CLL [257]. NCI reported responses in four CLL patients treated with CD19 CAR-T cells featuring CD28 costimulatory domain, including three complete remissions exceeding 1 year [256]. At MSKCC, seven evaluable CLL patients showed no responses to a different CAR-T design, possibly due to protocol differences or patient selection [212]. CD19 CAR-T therapy shows promise for relapsed/high-risk CLL, with some patients achieving durable remissions [248]. Further research is needed to improve response rates and understand outcome-influencing factors.

Implementing CAR-T therapy for AML poses distinct obstacles due to the similarity between malignant and healthy hematopoietic cell surface markers. The antigens commonly found on AML cells, including CD33, CD34, CD123, and CD135 (FLT3), are also expressed on normal bone marrow cells. Specifically, CD33 is detected on fully developed neutrophils, monocytes, and tissue macrophages, while CD123 appears on both lymphoid progenitors and certain differentiated lymphoid and myeloid cell populations. Evidence from CAR-T-19 clinical studies indicates that targeting these shared antigens with CAR-T therapy could result in the depletion of healthy myeloid cells. Research suggests that potent CD33-directed CAR-T cells might induce complete myeloid cell elimination (pan-myeloablation), and similar outcomes have been observed with CD123-targeted CAR-T cells in various studies [258, 259]. In experimental work conducted by our research group using non-obese diabetic severe combined immunodeficiency γ chain-deficient (NSG) mice implanted with human AML cells or healthy CD34+ cells, treatment comparisons between control T cells, anti-CD33 CAR-T cells, and anti-CD123 CAR-T cells revealed that while CAR-T cells effectively eliminated AML, they simultaneously destroyed normal hematopoietic cells. The development of targeted and effective CAR-T cell therapies for AML continues to be hindered by the lack of truly AML-specific surface antigens [260].

Novel approaches in AML treatment include the development of suicide switches and tandem CAR designs, which aim to address the risk of myeloid aplasia associated with targeting common antigens like CD33 and CD123. Suicide switches enable regulated elimination of CAR-T cells when severe toxicity occurs, utilizing systems such as inducible caspase-9 activation. Meanwhile, tandem CAR designs implement dual-targeting mechanisms to improve specificity and minimize collateral damage to healthy myeloid cells [261, 262].

Research has investigated various AML cell surface molecules as potential therapeutic targets, including CD33, CD123, CLL-1, CD70, and TIM-3. Although antibody-based therapies targeting these antigens have demonstrated potential in both animal studies and clinical trials, their therapeutic impact has been modest. Drawing from the remarkable success of CD19 CAR-T cell therapy in treating pediatric B-ALL, scientists have developed CAR-T cell approaches targeting AML-specific antigens. Preliminary research demonstrates that these CAR-T therapeutic strategies show greater anti-tumor effectiveness compared to antibody-based interventions.

### Anti-CD33 CAR-therapy for AML

Leukemia cells express a protein called CD33, which has been the focus of several therapeutic approaches [263–265]. While monoclonal antibodies like lintuzumab and ADCs such as gemtuzumab ozogamicin have shown some efficacy, they have notable limitations [266].

CD33-targeting CAR-T cell therapy has emerged as a viable option. Although effective, first-generation CAR-T cells have been associated with significant adverse effects [267–269]. However, second-generation CAR-T cells incorporating 4-1BB-CD3ζ signaling domains have demonstrated improved efficacy and reduced toxicity. These cells are capable of efficiently eliminating leukemia cells even at low effector-to-target ratios [259, 270–273].

CAR-T cell therapy for CD33-positive leukemias encounters obstacles such as treatment resistance and significant adverse effects, requiring additional research and refinement to enhance efficacy and reduce risks [274].

### Anti-CD123 CAR-therapy for AML

Research continues to identify optimal targets for acute myeloid leukemia therapy. The interleukin-3 receptor subunit, CD123, has emerged as a promising target due to its high expression levels on AML blast cells. Unlike certain myeloid markers, CD123 expression extends across various immune cell types, including monocytes, B cells, and dendritic cells [251].

Laboratory investigations of CD123-targeted CAR-T cells have yielded encouraging results, consistent with previous antibody-based therapy studies, showing minimal damage to healthy blood-forming cells [211, 213]. Research by Mardiros and colleagues demonstrated enhanced survival rates in animal studies using CD123-directed CAR-T cells with CD28 co-stimulation, though sustained effects were restricted, possibly attributed to retroviral vector usage [275].

Significant findings emerged from studies employing patient-derived AML xenografts (PDX) treated with CART123, which exhibited tumor destruction patterns and cytokine release syndrome similar to those seen in B-cell leukemia patients treated with CART19 cells [259].

CART123 effectively eliminated CD123^dim^ leukemic cells while establishing a T-cell memory pool that enabled rapid disease resistance. This contrasted with CART33 or CART12, which significantly reduced myeloid progenitors and blood stem cells in humanized mouse studies [276]. The observation that 70% of AML blasts express both CD33 and CD123 provides strong support for targeting these antigens simultaneously in AML treatment [265], leading to multiple ongoing clinical studies [274, 277]. While preclinical evidence strongly supports using CAR-T cells targeting both CD123 and CD33 for AML treatment, concerns remain about potential damage to healthy bone marrow and progenitor cells during therapy. However, the consistently higher expression of CD33 and CD123 on AML blasts compared to normal hematopoietic stem cells suggests potential therapeutic benefits with minimal impact on healthy bone marrow function [274].

Scientists propose developing bispecific CARs by combining CD33 and CD123 single-chain variable fragments with split CAR signaling domains. This approach has shown optimal cell multiplication, robust cytokine production, reduced toxicity toward healthy cells, and minimal impact on blood-forming stem cells [274].

### Anti-CLL-1 CAR-therapy for AML

C-type lectin-like molecule-1 (CLL-1) has emerged as a promising candidate for AML therapy, given its restricted expression on myeloid cells in the blood and bone marrow [258, 275]. Wang et al. engineered CAR-T cells that target CLL-1, which is predominantly absent in uncommitted stem cells but expressed in particular progenitor cells, thereby minimizing the likelihood of affecting healthy tissues [278]. These CAR-T cells successfully eliminated CLL-1-expressing AML cells in preclinical models, encompassing xenografted mice and co-cultures with healthy donor-derived hematopoietic stem cells. Significantly, CLL-1 CAR-T cells preserved healthy hematopoietic stem cells, in contrast to CAR-T cells directed against CD33 or CD123, which are also present on hematopoietic stem cells [278]. Tashiro et al. developed CLL-1-specific CAR-T cells (CLL-1.CAR-Ts) that showed targeted activity against CLL-1 + AML cell lines, patient samples, and AML xenografts in both in vitro and in vivo models. They added a “suicide switch” using a caspase-9 gene to ensure safety and reduce the risk of unintentionally depleting healthy myeloid cells [279]. One potential limitation of CLL-1 as a target is its variable expression on mature myeloid cells. However, the ability of CLL-1 CAR-T cells to spare normal progenitor cells, combined with the potential for regenerating mature myeloid cells after therapy, suggests this approach shows significant promise [279].

### Anti-h8F4-CAR-T therapy for AML

In the realm of CAR-T cell therapy for AML, researchers are actively exploring new potential targets. One promising candidate is the PR1 peptide, derived from leukemia-associated proteins, which is highly expressed on HLA-A2+ AML cells [280, 281].

Previous research has highlighted the potential of PR1-specific cytotoxic T lymphocytes (CTLs) in the treatment of AML. Molldrem and colleagues found that PR1-generated CTLs from healthy HLA-A2+ individuals can effectively target and destroy myeloid leukemia cells [280]. Similarly, PR1-specific CTLs identified in the blood of AML patients have shown the capability to eliminate leukemic blasts in vitro and reduce tumor burden in xenograft models [281, 282]. PR1-specific cytotoxic T lymphocytes have been associated with the graft-versus-leukemia (GVL) effect noted following allogeneic stem cell transplantation (allo-SCT) [281, 282].

Further research demonstrated that a humanized version of m84 (h8F4) retained its anti-cancer efficacy against AML, specifically targeting PR1/HLA-A2+ cells [283]. Umbilical cord blood (UCB) lymphocytes, characterized by their naïve T cell properties, are gaining recognition as promising candidates for CAR-T cell therapy [284–286]. Since umbilical cord blood transplantation (CBT) is already used to treat various diseases [285, 286], it offers a readily available source of T cells.

Researchers have effectively modified UCB T cells with the h8F4 CAR, enabling them to eradicate leukemic cells in a PR1/HLA-A2-dependent fashion [284]. Preliminary findings suggest that h8F4-CAR-T cell therapy holds promise as an innovative approach targeting a self-antigen specifically overexpressed on leukemic stem cells.

### Anti-CD70-directed CAR-T cells for AML

CD70 is emerging as a promising target for CAR-T cell therapy in AML. It is expressed on both the bulk AML cells and leukemia stem cells (LSCs), with little to no expression on HSCs [287]. This selective expression makes CD70 an attractive therapeutic target. Reither and colleagues discovered that the use of a hypomethylating agent (HMA) enhanced CD70 expression in AML LSCs [288]. Building on this finding, researchers investigated cusatuzumab, a human monoclonal antibody targeting CD70, designed to block the CD70/CD27 interaction and eliminate LSCs through antibody-dependent cell-mediated cytotoxicity (ADCC). Although preclinical studies using a PDX model showed promising results, a phase II clinical trial combining cusatuzumab with HMA yielded less favorable response rates compared to phase I [289, 290].

Sauer and colleagues recently demonstrated that CD70-specific CAR-T cells can effectively target AML while maintaining the integrity of HSCs. Their study evaluated various CAR designs and identified CD27z-CAR-T cells as having superior tumor-targeting activity and enhanced proliferation potential [291].

Research from Marcela Maus’ laboratory, presented at the 2021 American Society of Hematology (ASH) meeting, identified a soluble variant of the CD70 ligand, CD27, as a factor that could reduce the effectiveness of CD70-CAR-T cells in AML co-cultures [292]. To address this challenge, the team tested a range of novel hinge CAR variants. Among these, the CD8hinge&TM variant showed significantly improved cytolytic activity against AML targets in vitro compared to standard 4-1BB-based CAR-T cells. Additionally, CD8hinge&TM CAR-T cells demonstrated superior in vivo proliferation and more effectively eradicated AML in a PDX model compared to the original CAR-T cells. These findings suggest that CD70-CAR-T cell therapy, especially with hinge modifications to counteract the soluble ligand issue, holds promise for the treatment of AML [292].

### Anti-TIM-3 CAR-therapy for AML

Leukemia recurrence following chemotherapy is often driven by a small population of leukemic stem cells (LSCs) that are resistant to treatment and capable of proliferating and evolving into new leukemia blasts. Traditional therapies fail to eliminate these LSCs, allowing them to survive and cause relapse. Recent research has highlighted the potential role of T cell immunoglobulin mucin-3 (TIM-3) in the persistence of LSCs. TIM-3, an immune checkpoint molecule, is expressed in LSCs in most AML subtypes but is absent in healthy HSCs [291]. This selective expression makes TIM-3 an auspicious candidate for CAR-T cell therapy.

Research by Kikushige and colleagues demonstrated that only TIM-3+ AML cells, not TIM-3− cells, were capable of initiating leukemia in immunodeficient mice [293]. This finding underscores the critical role of TIM-3+ LSCs in the development of the disease. Targeting TIM-3 on LSCs offers a promising strategy to eradicate minimal residual disease (MRD) and enhance clinical outcomes for AML patients. TIM-3 is primarily expressed on AML blasts and LSCs, exhibiting minimal expression in normal tissues, thus rendering it a highly specific therapeutic target [294].

Wang and colleagues developed second-generation CAR-T cells targeting TIM-3 using antibodies derived from a human Fab phage library. These anti-TIM-3 CAR-T cells demonstrated strong antileukemic activity against AML cell lines, primary patient blasts, and in a xenograft murine model. The CAR-T cells effectively suppressed tumor growth and eradicated primary LSCs derived from patients. These findings suggest that administering anti-TIM-3 CAR-T cell therapy after initial treatment could significantly improve clinical outcomes for AML patients [294].

Further research by Xin He and associates explored bispecific and split CAR-T cells targeting both TIM-3 and CD13, an antigen highly expressed in LSCs [295]. Bispecific CAR-T cells successfully eradicated AML cells in murine models while reducing cytotoxicity to healthy stem cells and peripheral myeloid cells. These studies identify TIM-3 and CD13 as potential targets for CAR-T cell therapy in AML, with the capability to eliminate LSCs and avert disease recurrence [295].

Broadening the spectrum of CAR-T cell therapy targets can augment its versatility and enhance treatment outcomes for leukemia, potentially expanding its applicability to other cancer types and improving its clinical utility.

## Limitations of cancer vaccines and CAR-T cell therapies

### Cancer vaccines

While cancer vaccines show potential, numerous obstacles impede their broad implementation and success (Table 3). This analysis explores these impediments across different vaccine types and their impact on clinical application. For cell-based vaccines, individualized production requires substantial resources and incurs significant manufacturing costs, limiting accessibility through increased healthcare expenses. The immunosuppressive nature of tumor microenvironments often compromises the function of activated immune cells, reducing their cancer-fighting capability. Furthermore, cancer cells can develop resistance mechanisms against immune responses, undermining vaccine effectiveness and facilitating tumor progression. The stringent storage and transportation requirements create additional logistical challenges that may affect vaccine stability during distribution [296].

**Table 3:** Challenges of using cancer vaccines

Vaccine category	Challenges and limitations	References
Cell-based vaccines	• High-cost and resource-intensive production • Tumor-suppressive environment hinders immune cell function • Tumor evasion mechanisms can limit vaccine efficacy • Stringent storage and transport requirements	[296]
iPSC-based vaccines	• Ethical concerns regarding the use of embryonic stem cells and genetic alteration • Need for extensive preclinical safety testing • Potential for immune evasion similar to other vaccines	[297]
In situ vaccines	• Identifying tumor antigens in highly diverse cancers • Suppressive tumor microenvironment can reduce effectiveness • Limited to localized tumors, potentially ineffective against metastasis	[298, 299]
Viral vector vaccines	• Pre-existing immunity to vectors can reduce effectiveness • Safety concerns associated with some vectors • Limited cargo capacity restricts the number of antigens delivered	[300]
Nucleic acid vaccines (DNA or RNA)	• Need for specialized delivery systems for effective transfer • Transient antigen expression may require multiple doses • Development of immune tolerance can limit long-term efficacy	[301]
Peptide-based vaccines	• Target a limited number of antigens, neglecting others • Restricted to patients with compatible HLA types • Tumor diversity can render them ineffective against some cells • May elicit diminished immune responses relative to whole-cell vaccines • Often require adjuvants to enhance immunogenicity, increasing complexity	[302]
Exosome-based vaccines	• Complex optimization required for large-scale production • The effective loading of exosomes with tumor antigens is essential • Potential for unintended effects on immune response due to exosome modulation	[302]

The development of iPSC-based vaccines raises ethical concerns, particularly regarding embryonic stem cell utilization and genetic modifications. Thorough safety assessments must precede clinical implementation to address risks such as potential tumor formation or unexpected cellular responses. Like other cancer vaccines, these approaches must combat immune evasion strategies that can suppress anti-tumor immune responses [297].

In situ vaccination faces challenges in identifying specific tumor antigens, particularly in genetically complex cancers. The complex tumor environment can inhibit immune responses, diminishing vaccine performance. These vaccines may also show limited effectiveness against metastatic disease due to their primarily localized effects [298, 299].

The effectiveness of viral vector vaccines can be compromised by pre-existing immunity, especially in patients previously exposed to similar vectors. Vector safety requires careful evaluation to prevent adverse reactions. Additionally, these vectors' limited capacity for multiple tumor antigen delivery may restrict the breadth of immune responses [300].

For nucleic acid vaccines (DNA or RNA), sophisticated delivery systems are crucial for effective cellular uptake. The transient nature of antigen expression often necessitates multiple doses to maintain immune response strength. Potential development of immune tolerance to encoded antigens may limit long-term effectiveness, highlighting the need for enhanced durability strategies [301].

Peptide-based vaccines’ focus on specific tumor antigens might overlook other important targets, limiting comprehensive anti-tumor immunity. Their dependence on HLA compatibility restricts patient eligibility. Tumor heterogeneity poses additional challenges, as some cancer cells may lack targeted peptides. These vaccines typically generate weaker immune responses compared to whole-cell approaches, requiring additional immunogenic enhancement. The need for adjuvants increases formulation complexity [302].

Exosome-based therapeutic applications face challenges in developing efficient large-scale production methods to ensure adequate supply. Successful antigen loading is crucial for optimal immunogenicity and vaccine effectiveness. Their immune-modulatory properties could potentially impact vaccine efficacy and safety, emphasizing the importance of understanding their immune system interactions.

### CAR-T cell therapies

#### Antigen evasion

A significant challenge in CAR-T cell therapy is the development of tumor resistance (Table 4). While CAR-T cells initially demonstrate substantial efficacy by targeting specific antigens, many patients eventually experience a decrease or loss of the targeted antigen in their tumor cells—a phenomenon known as antigen escape. For instance, in relapsed/refractory ALL, 70–90% of patients initially respond to CD19-targeted CAR-T cell therapy. This issue is not limited to hematological cancers but also occurs in solid tumors. For example, in glioblastoma, a case study involving CAR-T cells targeting IL13Ra2 reported a decrease in IL13Ra2 expression in recurrent tumors [303].

**Table 4:** Challenges associated with CAR-T cell therapy

Challenge	How does it restrict using CAT-T cell therapy?	References
Antigen escape	Tumor cells can lose or reduce expression of the targeted antigen, rendering CAR-T cells ineffective	[303, 343–347]
Dual/tandem CAR strategies	Using CARs targeting multiple antigens to address antigen escape	[304–309]
On-target, off-tumor effects	CAR-T-cells may attack healthy tissues that express the targeted antigen	[310–312, 314]
CAR-T cell trafficking and tumor infiltration	Difficulty for CAR-T cells to reach and infiltrate solid tumors	[176]
Regional delivery	Strategy to deliver CAR-T cells directly to the tumor site	[317–319]
CAR-T cell modification	Engineering CAR-T cells to improve trafficking and infiltration	[320–322]
Stroma	Dense tissue network that hinders CAR-T cell infiltration	[323, 324, 348]
Immunosuppressive microenvironment	Tumors create an environment that suppresses immune response	[325, 326]
Checkpoint blockade	Combining CAR-T with drugs that block immune checkpoints to enhance persistence	[327, 328, 349]
Armored CARs	Engineering CAR-T cells to resist immunosuppression or secrete immunostimulatory signals	[273, 329–332]
CAR-T cell-associated toxicities	Significant adverse effects encompass CRS, HLH/MAS, and ICANS	[333–335]

Early clinical trials suggest that dual-targeted approaches may lead to more durable remissions. Current trials are exploring combinations such as CD19/CD20 and CD19/CD22 [188]. Early research with dual-targeted CAR-T cell approaches, particularly those combining CD19/CD22 and CD19/BCMA, has yielded encouraging outcomes [304–307]. Specifically, preliminary clinical trial results using CD19/CD22 CAR-T treatment in adult patients with ALL and diffuse large BCL have demonstrated positive outcomes [304–307]. Concurrent studies investigating BCMA/CD19 targeting in MM patients have revealed significant therapeutic effectiveness while maintaining acceptable safety levels [304, 305].

In the realm of solid tumors, laboratory investigations have explored various tandem CAR combinations, such as HER2/IL13Ra2 for glioblastoma treatment and HER2/MUC1 for breast cancer therapy. Both approaches demonstrated enhanced tumor-fighting capabilities compared to single-target treatments [191, 308]. Glioblastoma studies revealed that combining HER2 and IL13Ra2-targeted CARs resulted in improved anti-tumor activity and reduced antigen escape compared to other dual-targeting strategies [309]. These results emphasize the importance of strategic antigen selection to maximize tumor elimination while minimizing the risk of relapse through antigen escape mechanisms.

#### On-target off-tumor effects

The development of CAR-T therapy for solid tumors faces a fundamental obstacle: target antigens are often present not only on tumor cells but also on normal tissues at various levels. The design of effective CARs requires meticulous antigen selection to achieve optimal therapeutic benefits while reducing “on-target, off-tumor” adverse effects. One innovative approach involves targeting tumor-specific antigen modifications instead of the antigens themselves. For instance, modified *O*-glycans, including Tn and sialyl-Tn (STn), show high expression in certain solid tumors but are rarely found in healthy tissues [310]. Research has investigated numerous potential CAR-T targets for solid tumor treatment, including TAG72, B7-H3 [311, 312], MUC1 [313], and MUC16 [314, 315]. Early clinical studies of first-generation CAR-T cells targeting TAG72 in colon cancer showed limited therapeutic effect; current investigations focus on exploring next-generation targets and tumor-specific modifications [316]. The advancement of CAR-T therapy depends on developing novel approaches to overcome two key obstacles: preventing antigen escape and identifying targets that effectively eliminate tumors while minimizing damage to healthy tissues. Successfully addressing these challenges is essential for broadening CAR-T therapy applications across both blood cancers and solid tumors.

#### CAR-T cell trafficking and tumor infiltration

When comparing CAR-T cell therapy in solid tumors versus blood cancers, several unique obstacles emerge. Key challenges include navigating through the tumor's physical barriers and surviving in its hostile microenvironment. The dense stromal network surrounding solid tumors creates a physical obstruction, while the immunosuppressive tumor microenvironment (TME) diminishes CAR-T cell function and immune responses [176].

To combat these issues, researchers have explored direct tumor-site administration of CAR-T cells as one potential solution. This technique bypasses the need for stromal penetration and minimizes effects on healthy tissue. Promising results emerged from preclinical studies where HER2- or IL13Ra2-targeted CAR-T cells were directly introduced into brain tissue to treat glioblastoma and breast cancer brain metastases [317, 318]. These encouraging findings led to several clinical trials investigating local CAR-T delivery for brain tumors [NCT02208362, NCT03389230, NCT03696030]. Additionally, researchers are studying intrapleural administration for mesothelioma treatment through an ongoing phase 1 trial [NCT02414269] [319]. However, this localized approach may only be practical for patients with single tumors or limited metastatic spread [188].

Scientists are also developing methods to improve tumor infiltration and targeting by CAR-T cells. One innovative strategy involves modifying CAR-T cells to express specific chemokine receptors, essentially providing them with molecular navigation systems to follow tumor-produced chemokine signals. Enhanced tumor-homing abilities and anti-cancer effectiveness have been demonstrated in CAR-T cells expressing CXCR1, CXCR2, or integrin αvβ6 [320–322].

Another focus area involves addressing the stromal barrier. Studies have shown improved outcomes when CAR-T cells are engineered to produce heparanase, which degrades a major stromal component called heparan sulfate proteoglycan [323]. Furthermore, researchers have developed CAR-T cells targeting fibroblast activation protein (FAP), which is abundant in tumor stroma. These modified cells have demonstrated the ability to reduce tumor-associated fibroblasts and enhance anti-tumor responses in experimental models [324].

#### Immunosuppressive microenvironment

The future success of CAR-T cell therapy in treating solid tumors depends on creating new delivery systems and methods to enhance tumor penetration, which is crucial for broadening its use in treating complex solid malignancies and their spread [325]. The tumor environment contains various immunosuppressive elements, such as MDSCs, TAMs, and Tregs, which release substances that support tumor growth. The presence of immune checkpoint molecules like PD-1 and CTLA-4 creates additional barriers to effective immune responses [326].

Limited T cell growth and survival represent significant obstacles in CAR-T therapy, with co-inhibitory pathways potentially leading to T cell exhaustion [326]. Scientists are exploring the combination of CAR-T therapy with checkpoint inhibition as a promising strategy, utilizing CAR-T cells to target tumors while using PD-1/PD-L1 blockers to maintain T cell activity [327]. Research at the Children's Hospital of Pennsylvania demonstrated positive results in pediatric B-ALL patients when combining CD19 CAR-T therapy with PD-1 blockade [369]. Multiple clinical trials are currently investigating this dual approach for solid tumor treatment [328].

To combat the immunosuppressive tumor environment effectively, researchers are developing modified CAR-T cells that can withstand suppressive signals like TGF-β [329]. Another innovative strategy involves creating CAR-T cells that produce stimulatory cytokines to enhance their survival and anti-tumor effects [330]. Studies have investigated various “armored CARs,” including those that release IL-12 or express IL-15 [331], and those that convert immunosuppressive signals into pro-inflammatory responses [332]. Although combining checkpoint inhibition with CAR-T therapy shows potential, this approach alone may not guarantee successful T-cell infiltration and function in all cases. Additional research must explore how to integrate these approaches with other immunotherapy strategies, particularly for challenging blood cancers and solid tumors.

#### CAR-T cell-associated toxicities

While CAR-T cell therapy represents a revolutionary advancement in cancer treatment, its implementation as a primary treatment option remains limited due to significant adverse effects [333]. The spectrum of complications includes CRS, HLH/MAS, and ICANS, with their intensity varying based on factors including CAR design, target selection, and tumor type [333].

Our understanding of CAR-T cell-related complications stems primarily from studies of CD19-directed CAR-T cells, the pioneering FDA-approved treatment [176]. While clinical trials have shown remarkable response rates, they have also revealed potentially lethal complications [334]. In ALL/LBL treatment, nearly all patients experience some adverse reactions, with severe CRS affecting 23–46% of cases [335].

The heightened immune response and cytokine production associated with CAR-T therapy can trigger three primary complications. CRS, the first major toxicity, manifests through widespread cytokine release and T-cell expansion [336]. Initial symptoms may be mild, including fever and fatigue, but can escalate to severe complications such as cardiovascular collapse and multi-organ failure [337]. Studies show CRS affects 77–93% of leukemia patients and 37–93% of lymphoma patients receiving CAR-T therapy, with many experiencing severe manifestations [338, 339]. HLH/MAS, the second major complication, is difficult to distinguish from severe CRS but is estimated to affect approximately 1% of treated patients [336, 340]. ICANS, the third major toxicity, impacts the central nervous system, with manifestations ranging from mild cognitive changes to life-threatening cerebral edema [336, 340, 341]. While IL-6 blockers effectively treat CRS, ICANS typically requires corticosteroid intervention [249, 342].

Given the absence of preventive measures for these complications, research continues to focus on improving CAR design and developing strategies to minimize treatment-related toxicities [340]. Subsequent discussions will detail ongoing efforts to enhance both the safety profile and therapeutic efficacy of CAR-T cell therapy.

## Combining CAR-T cell therapy and cancer vaccines

### Rationale for combination therapies: synergistic mechanisms and benefits

**Fig. 6:**
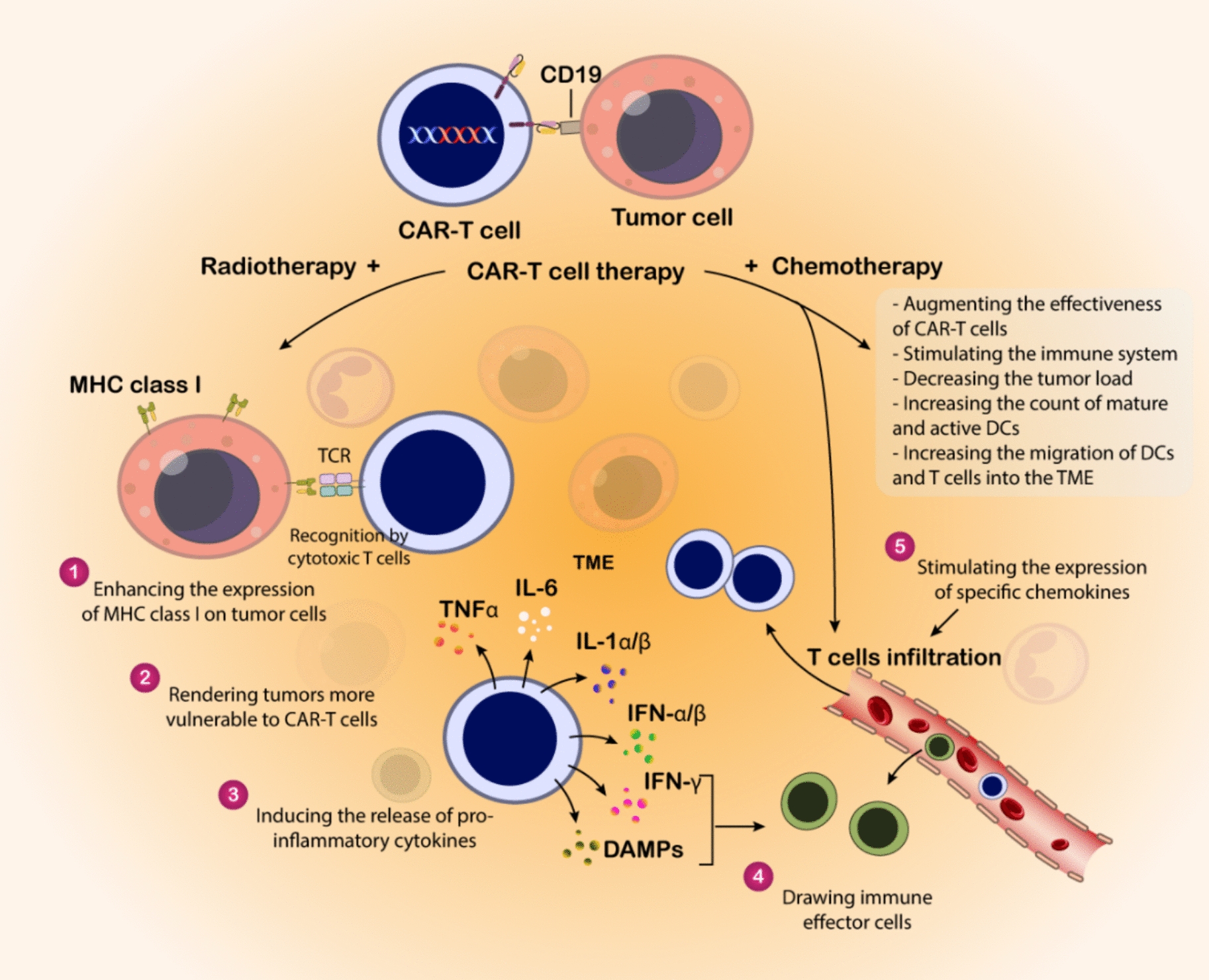
CAR-T cell combination therapies: mechanisms and benefits. As we can see, a CAR-T cell targeting a tumor cell expressing CD19. Prior or concurrent radiotherapy and chemotherapy are shown to exert several beneficial effects on the TME and the tumor cells themselves, thereby augmenting CAR-T cell efficacy. These effects include: (1) enhancing the expression of MHC class I molecules on tumor cells, potentially improving recognition by endogenous cytotoxic T cells; (2) rendering tumors more vulnerable to CAR-T cell-mediated killing; (3) inducing the release of pro-inflammatory cytokines (TNFα, IL-6, IL-1α/β, IFN-α/β, IFN-γ) and damage-associated molecular patterns (DAMPs) within the TME; (4) drawing immune effector cells, including CAR-T cells, into the TME; and (5) stimulating the expression of specific chemokines that further promote the infiltration of T cells into the tumor. Additionally, the combination therapy aims to directly augment the effectiveness of CAR-T cells, stimulate the broader immune system, decrease the overall tumor burden, increase the count of mature and active dendritic cells (DCs), and enhance the migration of DCs and T cells into the TME

Emerging research indicates that integrating CAR-T cell therapy with other therapeutic modalities offers promising opportunities to enhance cancer treatment efficacy, particularly for solid malignancies (as illustrated in Table 5 and Fig. 6). Research indicates that these integrated approaches can markedly improve CAR-T cell performance while minimizing adverse effects [350, 351]. Various therapeutic options can be combined with CAR-T cells, including traditional cancer treatments like chemotherapy and radiation, as well as newer approaches such as oncolytic viruses and cancer vaccines. These collaborative strategies work through multiple mechanisms to enhance treatment outcomes: reshaping the tumor environment, optimizing CAR architecture, improving tumor targeting precision, addressing tumor heterogeneity through multiple antigen targeting, countering immune escape mechanisms, and reducing treatment-related complications [352]. A notable example of successful combination therapy comes from BioNTech’s recent findings with their CLDN6-LPX protocol, which integrates CLDN6-targeted CAR-T cells with an innovative CAR-T cell-enhancing RNA vaccine (CARVac). Their Phase 1/2 clinical trial yielded impressive results, achieving a 59% overall response rate and a 95% disease control rate when CARVac was incorporated. These findings highlight the potential of this innovative approach in maintaining CAR-T cell activity and improving outcomes in patients with CLDN6-expressing solid tumors [353, 354].

**Table 5:** CAR-T cell therapies combined with cancer vaccines

Vaccine type	Description	Advantages	Examples	References
Cellular	Uses whole cells or cellular components as antigen sources and for presentation to APCs	• Can be modified to express costimulatory molecules	• K562 tumor cell line modified to synthesize immunogenic protein	[378, 380, 381]
		• DCs process and present antigens to T cells	• DC vaccines aim to stimulate long-term immune memory • DCs pulsed with specific tumor antigens to enhance CAR-T cell persistence	[383]
Molecular	Employs peptides, RNA, or DNA to deliver tumor antigens to APCs	• More economical than cellular vaccines	• CAROT cells (CAR + MHC-directed antigens) with OVA-Clec9A-TNE vaccine for sustained remission in mice	[382–384]
		• Challenge: Efficient delivery to APCs in lymph nodes	• Liposomal antigen-encoding RNA (RNA-LPX) for combination with CAR-T cells targeting claudin 6 (CLDN6)	
Viral vector	Employs whole viruses or viral antigens to activate virus-specific T cells	• Practical option for augmenting CAR-T cell therapy	• Bispecific T cells with CD19-CAR and CMVpp65 specificity for enhanced anti-tumor activity	[366, 394]
		• Modified vaccinia virus expressing human gp100 (VV-gp100) in conjunction with Her2-CAR-T cells (ACTIV therapy)	• Induces tumor regression through multiple mechanisms	
mRNA-based	Delivers mRNA encoding the CAR molecule to APCs	• Expands and persists adoptively transferred CAR-T cells	• RNA-lipoplex (RNA-LPX) to deliver CLDN6-CAR mRNA to APCs after CAR-T cell infusion	[361, 362, 368–378]
		• Targets tumors expressing CLDN6 and CLDN18.2 (low risk of off-target effects)	• Demonstrated efficacy in preclinical and phase 1/2 clinical trials

### Preclinical and clinical evidence: studies supporting combination approaches

**Fig. 7:**
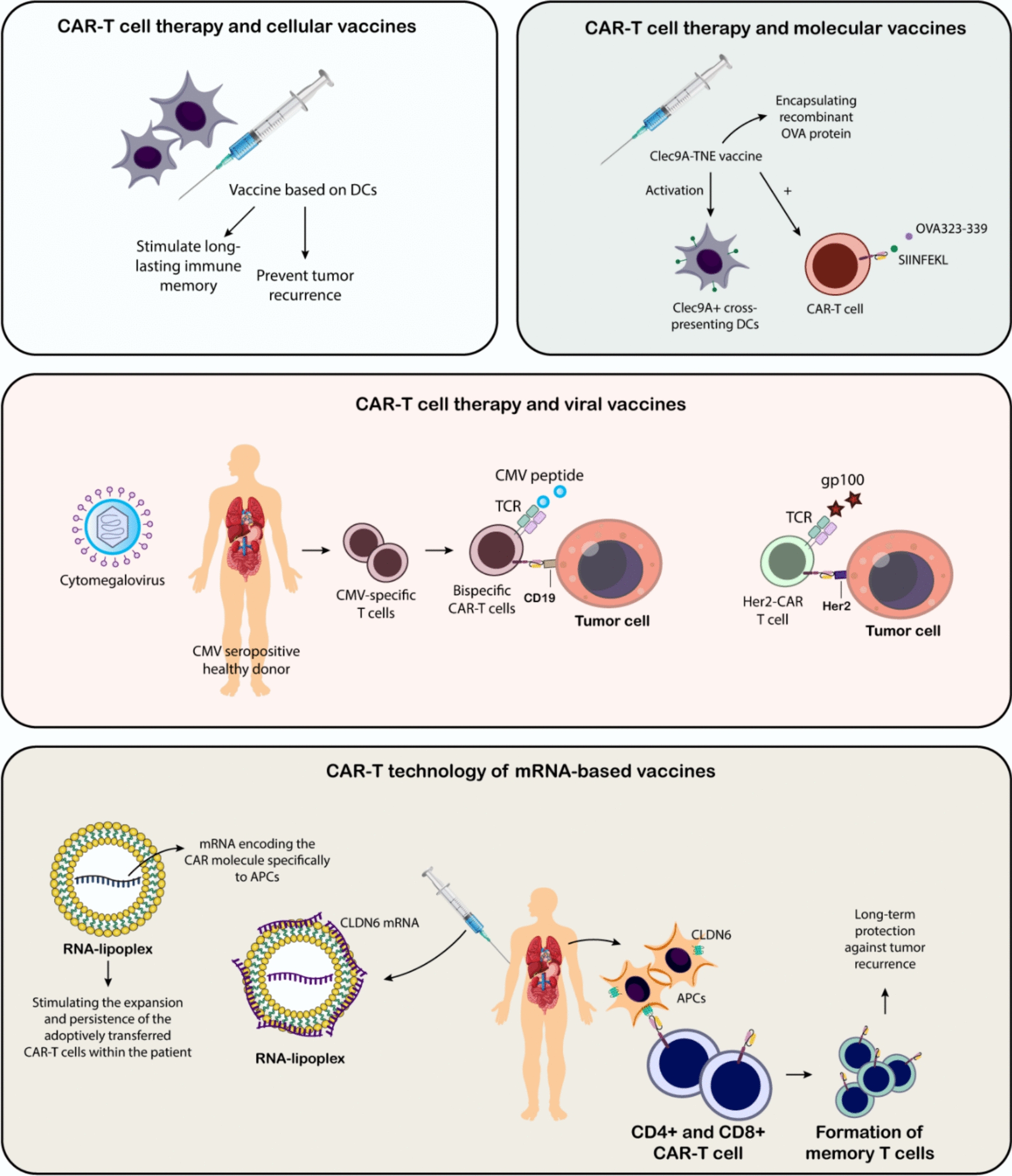
Integrated approaches combining CAR-T cell therapy with vaccine strategies. The top left panel depicts the combination of CAR-T cell therapy with cellular vaccines, specifically dendritic cell (DC)-based vaccines, aimed at stimulating long-lasting immune memory. The top right panel shows the integration of CAR-T cell therapy with molecular vaccines, where a Clec9A-TNE vaccine encapsulating recombinant OVA protein is used to activate Clec9A+ cross-presenting DCs, which then present tumor-associated antigens (e.g., SIINFEKL, OVA323-339) to further boost the CAR-T cell response. The middle panel explores the synergy between CAR-T cell therapy and viral vaccines, utilizing CMV-specific T cells from CMV-seropositive donors that can be engineered into bispecific CAR-T cells targeting both CMV peptides and tumor antigens (e.g., CD19). Similarly, Her2-CAR-T cells can be combined with vaccines targeting other tumor-associated antigens like gp100 to broaden the anti-tumor response. Finally, the bottom panel highlights the CAR-T technology of mRNA-based vaccines, where mRNA encoding the CAR molecule is specifically delivered to antigen-presenting cells (APCs) via RNA-lipoplexes. This approach, exemplified by CLDN6 mRNA delivery, aims to stimulate the expansion and persistence of adoptively transferred CAR-T cells within the patient, leading to the formation of memory T cells and long-term protection against tumor recurrence

Integrating CAR-T cell therapy with cancer vaccines represents an innovative therapeutic approach to tackle major obstacles in CAR-T treatment, particularly antigen loss and T-cell exhaustion [59]. As depicted in Fig. 7, cancer vaccines utilize tumor-associated antigens (TAAs) to stimulate specific immune responses, functioning as immune modulators to help overcome certain CAR-T therapy limitations. This combined strategy enhances CAR-T cell performance through two distinct pathways: indirect activation through antigen-presenting cells (APCs) or HLA-dependent mechanisms, and direct stimulation of dual- or bi-specific CAR-T cells within the tumor environment [60]. Cancer vaccines are classified into three fundamental categories according to their composition: cellular-based, molecular-based, and viral-based approaches [12].

#### CAR-T cell therapy and cellular vaccines

The integration of cellular vaccines with CAR-T cell therapy offers significant benefits, utilizing complete cells or their components as both antigen sources and delivery mechanisms to antigen-presenting cells (APCs). These vaccines can be derived from various sources, including tumor cells, radiation-treated cell lines, and dendritic cells [355]. Early investigations utilized modified K562 tumor cells expressing highly immunogenic proteins to combine CAR-T cells with cellular vaccines, enabling efficient viral epitope presentation to APCs [356]. Scientists developed cytomegalovirus (CMV)-specific cytotoxic T lymphocytes (CTLs) targeting both GD2 and CMV, combined with a cellular vaccine created from irradiated lymphoblastoid cells, which enhanced CAR-T cell longevity and multiplication in living organisms [357]. Dendritic cells (DCs), which are crucial for antigen processing and T cell presentation, serve as fundamental components of immune response. Current research explores DC-based vaccines to establish long-lasting immune memory and prevent cancer recurrence after CAR-T treatment [358]. As an example, Wu and colleagues created a vaccine using DCs exposed to specific tumor antigens, which substantially enhanced CAR-T cell survival and effectiveness in laboratory studies [359].

#### CAR-T cell therapy and molecular vaccines

Molecular vaccines, utilizing peptides, RNA, and DNA, represent a cost-efficient alternative to cellular vaccines when integrated with CAR-T cell therapy. Researchers are enhancing CAR-T cell efficacy by targeting tumor cells through MHC-presented antigens activated by the vaccine. Nonetheless, the effective delivery to antigen-presenting cells in lymph nodes poses a challenge, resulting in the creation of Nanoparticulate vaccine platforms [360]. Researchers employed ovalbumin peptides within a Clec9A-targeted Nano emulsion to deliver the OVA antigen to APCs. CAROT cells were engineered to activate OVA-specific T-cell receptors or HER2-specific chimeric antigen receptors upon interaction with HER2-positive neoplastic cells. The OVA-Clec9A-TNE vaccine resulted in prolonged solid tumor remission in mice, attributed to CAROT cell proliferation, anti-tumor effectiveness, and enduring immune memory [360].

Reinhard and colleagues investigated a novel therapeutic strategy combining nanoparticle-based vaccination with CAR-T cell treatment [361]. Their approach involved creating RNA-LPX by combining negatively charged mRNA with cationic liposomes. This RNA construction serves dual purposes: acting as an adjuvant to activate antigen-presenting cells through TLR pathways, and providing antigens to enhance dendritic cell presentation [362]. The study focused on CLDN6 as the target antigen for both CAR-T cells and vaccine-mediated dendritic cell presentation. This integrated approach, named CARVac, demonstrated improved CAR-T cell multiplication and killing efficiency in living subjects, leading to enhanced tumor control. These results suggest a practical, cost-effective, and scalable vaccination strategy to address current limitations of CAR-T cell therapy in solid tumors [362].

#### CAR-T cell therapy and viral vaccines

Viral vector vaccines employing complete viruses or specific viral antigens to stimulate virus-targeting T cells offer a feasible and encouraging approach for improving CAR-T cell therapy, as shown by multiple research studies [363–365].

One method involves creating T cells with both a CAR-Targeting tumor antigens and a TCR that recognizes viral antigens. Wang and colleagues developed CD19-CAR-T cells that could also be activated by CMVpp65 protein, enabling them to identify CMV peptides through their TCR in an HLA-dependent fashion [366]. Their research demonstrated that CMVpp65 vaccination enhanced the anti-tumor effectiveness of these dual-specificity T cells, indicating potential benefits for patients with inadequate responses to standard CAR-T therapy. This strategy could potentially be extended to various cancer types [366].

In a parallel investigation, Slaney’s team explored an alternative approach using a modified vaccinia virus expressing human gp100 (VV-gp100) in conjunction with Her2-targeting CAR-T cells [367]. Their ACTIV therapy protocol, which combined dual-specific T cells with recombinant vaccinia virus and high-dose IL-2, showed remarkable improvements in dual-specific T cell expansion. Unlike the CMV peptide vaccine, this approach provided partial protection against tumor recurrence in mouse models following initial tumor clearance [367].

#### CAR-T technology of mRNA-based vaccines

The advent of mRNA vaccines has opened new avenues in cancer therapy. These vaccines deliver mRNA molecules that direct cells to synthesize specific proteins [368]. This technology played a crucial role in the development of mRNA vaccines for COVID-19, showcasing its versatility and potential for treating various diseases [369].

In cancer immunotherapy, mRNA vaccines can stimulate the immune system to attack tumors. These vaccines are internalized by APCs, which subsequently present tumor-specific antigens to T cells, eliciting an immune response against cancer cells [362, 370, 371]. Researchers at BioNTech have proposed an innovative approach combining mRNA vaccines with ACT to improve the effectiveness of CAR-T cell therapy for solid tumors. This strategy aims to amplify CAR-T cell expansion, extend their lifespan, and enhance their antitumor activity [361].

The approach utilizes a delivery system called RNA-lipoplex (RNA-LPX) to deliver mRNA encoding the CAR molecule directly to APCs after CAR-T cell infusion. This process promotes the expansion and persistence of the transferred CAR-T cells within the patient. Intravenous administration of RNA-LPX to target lymphoid organs represents a promising strategy for cancer vaccines [372, 373].

The mRNA vaccine platform provides several advantages. Unlike amphiphilic ligand CAR-T technology, which relies on an amphiphilic platform to deliver ligands to DCs and is susceptible to degradation by extracellular enzymes, the mRNA-based approach avoids this vulnerability. Additionally, the CAR and vaccine target CLDN6 and CLDN18.2, which are commonly found in ovarian, testicular, endometrial, and gastric cancers [374, 375].

Notably, CLDN6 is absent in normal human tissues, minimizing the risk of off-target effects and autoimmune reactions [375, 376]. These studies confirm earlier findings that anti-CLDN6 does not cross-react with other claudin family proteins, further reducing the likelihood of unintended immune responses [377].

Preclinical studies in mice revealed a marked increase in proliferating CLDN6-CAR-T cells following treatment with CAR-CLDN6 and subsequent administration of CLDN6-LPX, resulting in complete tumor regression within 14 days. The approach also demonstrated effectiveness against tumors expressing CLDN18.2 and CD19, highlighting its potential for treating both solid and liquid tumors. Additionally, the treatment induced a significant proliferation of CD4+ and CD8+ CAR-T cells, suggesting the development of memory T cells capable of providing long-term protection against tumor recurrence. Early clinical trials (phase 1/2) have shown promising outcomes, with ovarian cancer patients receiving this combination therapy achieving an overall response rate of 43% and a disease control rate of 86% [378]. Likewise, patients with testicular cancer demonstrated an impressive response at a particular dose level. As of January 2022, 16 patients achieved complete remission, with side effects that were transient and manageable. These results highlight the potential of combining CAR-T cell therapy with mRNA vaccines as a promising approach for treating various cancers [379].

### Perspectives on vaccine-based CAR-T therapy

The main goal of CAR-T cell vaccine development is to extend their in vivo half-life and attain prolonged remission in solid cancer patients, despite the challenging tumor microenvironment, with vaccines potentially enhancing CAR-T cells and augmenting their antitumor effectiveness [379].

The TME in solid tumors hinders CAR-T cell infiltration, limiting their access to the tumor core and preventing their ability to eliminate cancer cells, potentially leading to post-CAR-T relapses [380–382]. Combining CAR-T cell therapy with booster vaccines offers a potential solution by enhancing the proliferation and longevity of CAR-T cells, potentially eliminating all tumors, including relapsed ones. Unlike earlier approaches that utilized immunogenic virus-specific T cells, modern CAR-T vaccines incorporate humanized antigens, which may trigger a less vigorous immune response. A significant advantage of this approach is the ability to administer a smaller dose of CAR-T cells, thereby reducing production costs and shortening the time required for in vitro CAR-T cell expansion.

Although mRNA vaccine-based CAR-T therapy shows potential for treating relapsed or refractory cancers, it is essential to account for the two distinct types of relapses in CAR-T immunotherapy [383, 384]. Positive relapses occur when tumor antigens persist, allowing existing CAR-T cells to identify and eliminate them. In contrast, negative relapses arise when tumor antigens are lost or downregulated, enabling tumors to evade detection and escape [384]. Vaccine-induced persistence of CAR-T cells can help address positive relapses but may fail against negative relapse tumors. Furthermore, tumor evolution driven by genomic instability and natural selection can undermine the effectiveness of CAR-T cells, even with prolonged persistence [385, 386]. Bivalent vaccine candidates simultaneously target two tumor antigen variants, potentially generating broader immunity against other variants. Engineered CAR-T cells can differentiate into various antigen-specific lymphocytes, targeting a broader range of tumors.

The effectiveness of antigen presentation by DCs varies depending on their maturation state. Immature DCs are adept at capturing tumor antigens but may have limited mobility, whereas mature DCs are highly effective at presenting antigens to T cells in lymph nodes but show a reduced capacity for antigen uptake. In certain solid tumors, such as breast cancer, DCs that appear mature often display features of immature DCs, resulting in compromised T cell activation [387]. Dysfunctional DCs can impair CAR-T cell activation by exhibiting reduced antigen presentation capabilities [388]. Researchers have recently demonstrated the repurposing of living tumor cells to home in on tumor sites, where they release antitumor agents and immune-stimulatory factors, leading to tumor eradication and long-lasting immunity [389]. Engineered living tumor cells show promise as vaccine candidates for activating CAR-T cells and promoting sustained antitumor immunity. Future research should focus on developing vaccines that incorporate bispecific CAR-T cell engagers specifically designed to target DCs, aiming to improve the clinical effectiveness of vaccine-based CAR-T therapy. The recognition and binding of T cells to tumor-associated MHC molecules on DCs are critical for T cell activation, clonal expansion, and tumor cell destruction. However, various inhibitory and immune tolerance mechanisms can hinder the interaction between CAR-T cells and DCs [390–393]. To address this, a bispecific T cell engager (BiTE) vaccine construct could be designed to strengthen this interaction. Similar to the current BiTE approach, which directs T cells to CD33-positive tumor cells, the BiTE vaccine would recruit CAR-T cells to lymph nodes, enabling them to quickly identify and bind to DCs, thereby enhancing their activation and efficacy [393].

## Future directions

Various immunotherapy strategies have shown promise in treating hematologic malignancies (blood cancers), each with its advantages and limitations. Further research is essential to improve clinical outcomes for patients [395].

Allo-HSCT is the most efficacious treatment for specific hematological malignancies, providing a potential cure. Haploidentical HSCT (haploid-HSCT) addresses the issue of a limited donor pool; however, challenges remain in reducing GVHD and transplant-related mortality while enhancing anti-tumor effects, particularly in relapsed/refractory cancers. The future of transplantation lies in personalized approaches, often involving combination therapies. A promising strategy involves employing CAR-T therapy or other targeted treatments to achieve remission in relapsed/refractory patients before hematopoietic stem cell transplantation (HSCT). Moreover, allo-HSCT, in conjunction with donor-derived CAR-T cells, presents a promising option for patients exhibiting residual disease following previous therapies. For individuals with minimal residual disease (MRD) post-transplantation, CAR-T therapy may offer considerable advantages [396, 397].

An alternative approach involves refining donor selection, particularly among family members, to exclude those who share the patient’s genetic abnormalities. For patients requiring a second transplant, molecular testing can identify HLA loss and guide the donor search process. Personalized post-transplant care is equally vital, focusing on balancing anti-tumor efficacy with GVHD prophylaxis. While monoclonal antibodies (mAbs), bispecific antibodies (bsAbs), and ADCs have shown significant promise in cancer treatment, patient responses and overall clinical outcomes remain limited. Identifying the most effective tumor antigens for antibody-based therapies is crucial to improving anti-tumor effects and minimizing off-target toxicities. Developing bsAbs is particularly challenging compared to mAbs, requiring a rational structural design informed by receptor architecture and disease biology. Selecting the ideal target combination is just the beginning of this intricate process. Improper dosage schedules and poorly designed clinical trials can lead to increased patient toxicity. Optimizing treatment plans, dosages, and schedules is key to minimizing side effects. In ADCs, the payload and linker play a crucial role in determining both effectiveness and safety. Ongoing research focuses on addressing medication resistance, enhancing efficacy, stabilizing drugs, and managing pharmacokinetic complexities. Developing bispecific antibody–drug conjugates (bsADCs), which combine the advantages of bispecific antibodies (bsAbs) and ADCs, presents a significant challenge. Compared to monoclonal antibodies (mAbs), bsADCs offer improved safety, greater tumor cell selectivity, and the potential to overcome drug resistance [1, 398].

To improve efficacy while ensuring safety, innovative therapeutic approaches are being developed, including bifunctional checkpoint inhibitors engaging T cells (CiTE), simultaneous multiple interaction T cell engagers (SMITE), trispecific killer engagers (TriKE), and BiTE-expressing CAR-T cells. These advanced agents integrate multiple immune functions into a single molecule or cellular vector. ICIs have demonstrated moderate success in treating various hematologic malignancies, with notable effectiveness in Hodgkin lymphoma and primary mediastinal large BCL. However, their clinical use can be limited by significant immune-related adverse events (irAEs). A key area of research focuses on identifying more rational and efficient combination strategies to enhance the efficacy of ICI therapy [1].

The emergence of Adoptive Cell Transfer (ACT), especially CAR-T-cell therapy, represents a significant breakthrough for patients with resistant or recurring hematological malignancies. While these treatments have shown impressive results, they face considerable obstacles, including severe complications like Cytokine Release Syndrome (CRS), ICANS, and unintended toxicities. Several challenges persist, such as the need to enhance response rates and longevity, decrease adverse effects, refine CAR engineering and manufacturing processes, and broaden the therapy's applications to diverse cancer types. Addressing tumor resistance and improving CAR-T cell effectiveness and survival in living organisms requires the development of novel approaches. Critical to this effort is comprehending how tumors evade immune responses and develop resistance. Epigenetic mechanisms significantly influence both immune system regulation and cancer development. Therapeutic agents targeting epigenetic modifications, including histone deacetylase inhibitors and DNA methylation inhibitors, show promise through their diverse mechanisms of action in improving treatment outcomes [399–401].

A deeper understanding of epi-immunotherapy is essential to fully realize its potential. Allogeneic CAR-T cells, or “off-the-shelf” CAR-T cells, present a promising alternative to the limitations of autologous CAR-T therapy. Although advancements in gene editing technologies are helping to address safety concerns, substantial challenges persist in creating effective and widely accessible universal CAR-T cell therapies [1].

Although immunotherapy is often reserved as a last-resort treatment, its growing effectiveness and improved safety profile indicate a future where it could become a first-line therapy, potentially decreasing the reliance on chemotherapy. Combining immunotherapy with chemotherapy provides a synergistic approach, offering enhanced therapeutic benefits [402].

Combination immunotherapy offers great potential for improving the treatment of hematologic malignancies. While HSC transplantation (HSCT) remains a foundational therapy, integrating it with emerging immunotherapies presents exciting opportunities. However, critical questions about the optimal timing and sequencing of these treatments remain unanswered. As technology continues to evolve, personalized immunotherapy approaches are expected to play an increasingly vital role, allowing for more precise targeting of cancer cells while minimizing side effects.

Overall, challenges and future directions in immunotherapy encompass a range of critical issues from reducing manufacturing costs by optimizing CAR-T cell production and developing allogeneic “off-the-shelf” solutions to streamline the logistics of personalized treatments like CAR-T therapy and HSCT. Toxicity management remains crucial, with efforts focused on mitigating severe side effects such as GVHD, CRS, ICANS, and off-target effects through better dosing and innovative therapeutic designs. Thus, enhancing efficacy and managing resistance are also vital, necessitating improvements in remission rates and the durability of treatments by addressing tumor resistance and enhancing therapeutic persistence.

Further challenges include the identification of effective tumor antigens for antibody-based therapies, development of complex therapeutic agents such as bsAbs, ADCs, and multi-specific T cell engagers, and optimization of combination therapies that integrate immunotherapies with traditional approaches like chemotherapy. Personalized treatment strategies need further development to tailor therapies to individual patient profiles. Advances in understanding immune escape mechanisms, the role of epi-immunotherapy, and cancer vaccine development are also essential. Additionally, there is a need to improve the design of clinical trials to more accurately assess the efficacy and safety of emerging immunotherapies, ensuring that new treatments can be effectively translated into clinical practice.

## Conclusion

Leukemias pose significant health challenges, but immunotherapy offers promising solutions through various strategies aimed at boosting the immune system's ability to fight cancer. Despite advancements, issues such as toxicity and resistance remain prevalent. CAR-T cell therapy shows great potential, but its effectiveness must be enhanced by addressing challenges like antigen loss and cellular exhaustion. Combining CAR-T cells with other treatments, such as chemotherapy or alternative immunotherapies, may help overcome resistance and improve overall effectiveness. Future research should focus on developing safer, more efficient CAR-T cells, understanding resistance mechanisms, and exploring innovative combination therapies to improve patient outcomes. Broad clinical implementation of combination immunotherapies for leukemias faces several translational hurdles. Firstly, the cost of CAR-T cell therapy, along with the complexity of manufacturing and delivering personalized treatments, poses a significant challenge for widespread adoption. Secondly, predicting and managing toxicities, such as CRS and neurotoxicity, requires further research to establish standardized protocols and risk-stratification strategies. Additionally, long-term efficacy and the potential for late relapses need to be addressed through extended follow-up studies and the development of strategies to enhance CAR-T cell persistence and overcome antigen escape. Finally, while combination therapies hold promise, identifying the optimal combinations, sequencing, and dosing regimens will require extensive clinical trials. If these challenges are adequately addressed, combination immunotherapies could become a standard treatment option for leukemia within the next 5–10 years.

## Data Availability

No datasets were generated or analysed during the current study.

## Abbreviations

ALL: Acute lymphoblastic leukemia
AML: Acute myeloid leukemia
ACTIV: Adoptive Cell Transfer Incorporating Vaccination
ADCC: Antibody-dependent cell-mediated cytotoxicity
ADCs: Antibody–Drug Conjugates
APCs: Antigen-presenting cells
BCG: Bacillus Calmette-Guérin
BCLs: B-cell lymphomas
BsAbs: Bispecific antibodies)
BiTE: Bispecific T-cell engager
CEA: Carcinoembryonic antigen
CiTE: Checkpoint inhibitors engaging T cells
CAR -T: Chimeric antigen receptor T cell
CLL: Chronic lymphocytic leukemia
DCs: Dendritic cells
DLBCL: Diffuse large B-cell lymphoma
ELISPOT: Enzyme-linked immunosorbent spot
EBV: Epstein-Barr virus
FDA: Food and Drug Association
FL: Follicular lymphoma
GM-CSF: Granulocyte–macrophage colony-stimulating factor
GVHD: Graft-versus-host disease
HLH/MAS: Hemophagocytic Lymphohistiocytosis/Macrophage Activation Syndrome
HLA: Human leukocyte antigen
HL: Hodgkin lymphoma
HSCT: HSC transplantation
RHAMM: Hyaluronic acid-mediated motility
HMA: Hypomethylating agent
ICIs: Immune checkpoint inhibitors
ICANS: Immune Effector Cell-Associated Neurotoxicity Syndrome
ICS: Intracellular cytokine staining
LSCs: Leukemia stem cells
LBL: Lymphoblastic lymphoma
MHC: Major histocompatibility complex
MUC1: Mucin 1 protein
MM: Multiple myeloma
MDS: Myelodysplastic syndromes
MDSCs: Myeloid-derived suppressor cells
NK: Natural killer cells
NHL: Non-Hodgkin lymphoma
PBMCs: Peripheral blood mononuclear cells
PD-1: Programmed cell death protein 1
PD-L1: Programmed death-ligand 1
PR3: Proteinase 3
SMITE: Simultaneous multiple interaction T cell engagers
TriKE: Trispecific killer engagers
TLRs: Toll-like receptors
TME: Tumor microenvironment
TMB: Tumor mutational burden
TAAs: Tumor-associated antigens
TSAs: Tumor-specific antigens
WT1: Wilms' Tumor 1
